# Embryonic expression patterns and phylogenetic analysis of panarthropod sox genes: insight into nervous system development, segmentation and gonadogenesis

**DOI:** 10.1186/s12862-018-1196-z

**Published:** 2018-06-08

**Authors:** Ralf Janssen, Emil Andersson, Ellinor Betnér, Sifra Bijl, Will Fowler, Lars Höök, Jake Leyhr, Alexander Mannelqvist, Virginia Panara, Kate Smith, Sydney Tiemann

**Affiliations:** Uppsala University, Department of Earth Sciences, Palaeobiology, Villavägen 16, 75236 Uppsala, Sweden

**Keywords:** Sry, Germ cells, Vasa, Arthropoda, Onychophora, Development

## Abstract

**Background:**

Sox (Sry-related high-mobility-group box) genes represent important factors in animal development. Relatively little, however, is known about the embryonic expression patterns and thus possible function(s) of Sox genes during ontogenesis in panarthropods (Arthropoda+Tardigrada+Onychophora). To date, studies have been restricted exclusively to higher insects, including the model system *Drosophila melanogaster*, with no comprehensive data available for any other arthropod group, or any tardigrade or onychophoran.

**Results:**

This study provides a phylogenetic analysis of panarthropod Sox genes and presents the first comprehensive analysis of embryonic expression patterns in the flour beetle *Tribolium castaneum* (Hexapoda), the pill millipede *Glomeris marginata* (Myriapoda), and the velvet worm, *Euperipatoides kanangrensis* (Onychophora). 24 Sox genes were identified and investigated: 7 in *Euperipatoides*, 8 in *Glomeris*, and 9 in *Tribolium*. Each species possesses at least one ortholog of each of the five expected Sox gene families, B, C, D, E, and F, many of which are differentially expressed during ontogenesis.

**Conclusion:**

Sox gene expression (and potentially function) is highly conserved in arthropods and their closest relatives, the onychophorans. Sox B, C and D class genes appear to be crucial for nervous system development, while the Sox B genes *Dichaete* (*D*) and *Sox21b* likely play an additional conserved role in panarthropod segmentation. The Sox B gene *Sox21a* likely has a conserved function in foregut and Malpighian tubule development, at least in Hexapoda. The data further suggest that Sox D and E genes are involved in mesoderm differentiation, and that Sox E genes are involved in gonadal development.

The new data expand our knowledge about the expression and implied function of Sox genes to Mandibulata (Myriapoda+Pancrustacea) and Panarthropoda (Arthropoda+Onychophora).

**Electronic supplementary material:**

The online version of this article (10.1186/s12862-018-1196-z) contains supplementary material, which is available to authorized users.

## Background

Sox (Sry-related high-mobility-group box) genes (Gubbay et al. [1]) encode transcription factors that are essential in animal developmental processes, such as neurogenesis (reviewed in Reiprich and Wegner [2], Neriec and Desplan [3]), mesoderm differentiation (e.g. Chimal-Monroy et al. [4], McCauley et al. [5]), and gonadogenesis (e.g. Nanda et al. [6], Jiang et al. [7]). Sox genes are subdivided into 10 groups. Group A contains only *Sry* which is specific to placental mammals. Sox class B, C, D, E and F are common to Metazoa. Sox classes G, H, I and J are lineage specific: *SoxG* is vertebrate specific, *SoxH* is specific for humans, *SoxI* is only found in the frog *Xenopus laevis*, and *SoxJ* is specific for the nematode *Caenorhabditis elegans* (Bowles et al. [8], Ito et al. [9]).

Despite their ubiquity among metazoans and their acknowledged importance for key developmental processes, Sox genes have not been investigated in any arthropod other than the vinegar fly *Drosophila melanogaster* (Cremazy et al. [10], McKimmie et al. [11]), or even in any other ecdysozoan species with the exception of *Caenorhabditis elegans* (Vidal et al. [12]). Moreover, even gene content and embryonic expression patterns have scarcely been investigated, excluding a study on honey bee Sox genes (*Apis mellifera*, Wilson and Dearden [13]) and studies presenting the embryonic expression patterns of a single Sox gene in the beetle *Tribolium castaneum* (Oberhofer et al. [14], Clark and Peel [15]) and the millipede *Glomeris marginata* (Pioro and Stollewerk [16]). A study investigating content and expression of Sox genes in the common house spider *Parasteatoda tepidariorum* has been submitted to BMC Evolutionary Biology (Paese et al. [17]).

In order to obtain a better understanding of the role that Sox genes play in panarthropod development and evolution, the complement of Sox genes and their embryonic expression patterns in the red flour beetle *Tribolium castaneum* (Hexapoda), the pill millipede *Glomeris marginata* (Myriapoda), and the velvet worm *Euperipatoides kanangrensis* (Onychophora), were investigated and compared with the limited existing arthropod Sox gene data, including the very recent data on the spider (Paese et al. [17]). As a representative of the Coleoptera, the work on *Tribolium* provides insight into gene expression (and implied function) between the evolutionary branches leading to the honey bee (*Apis mellifera*; Hymenoptera) and *Drosophila* (Diptera) (Savard et al. [18]). Additionally, the observations from *Glomeris* expand our knowledge beyond the hexapods to Mandibulata, whilst the new data from *Euperipatoides* elucidate Sox gene expression/function in a closely related arthropod outgroup (Campbell et al. [19], Borner et al. [20], Smith et al. [21]). Taken together, the results of this study thus give the first comprehensive comparative analysis into Sox gene expression (and implied function) in Panarthropoda. It was discovered that Sox B family genes are predominantly expressed in the nervous system and unlike the situation in the model arthropod *Drosophila*, so are Sox C family genes. A function of *Dichaete* orthologs during segmentation is likely conserved in panarthropods, whilst Sox E genes are probably involved in mesoderm specification and gonadogenesis.

## Methods

### Gene cloning

Total RNA was isolated from mixed embryonic stages of the beetle *Tribolium castaneum*, the millipede *Glomeris marginata*, and the onychophoran *Euperipatoides kanangrensis* using TRIZOL (Invitrogen, Carlsbad, CA, USA), and reverse transcribed into cDNA (SuperscriptIII first strand synthesis system, Invitrogen) for polymerase chain reaction (RT-PCR). Gene fragments were amplified using gene specific primers based on the sequences found in sequenced embryonic transcriptomes for *Glomeris* and *Euperipatoides* (Janssen and Posnien [22], Janssen and Budd [23]), and a published sequenced genome for *Tribolium* (*Tribolium* Genome Sequencing Consortium [24]). Primer sequences are provided in Additional file 1: Table S1. All fragments were Topo-TA cloned into the pCRII vector (Invitrogen), and their sequences were checked from both strands on an ABI3730XL analyser using Big Dye dye-terminators by a commercial sequencing service (Macrogen, Korea). Sequence data have been made public via submission to the European Nucleotide Archive (ENA). Accession numbers of all recovered gene fragments are summarized in Additional file 2: Table S2.

### Sequence analysis

Conserved amino acids of the SOX box were aligned using ClustalX with default parameters in MacVector v12.6.0 (MacVector, Inc., Cary, NC) (Additional file 3: Figure S1).

A phylogenetic analysis was performed with MrBayes (Huelsenbeck and Ronquist [25]) using a fixed WAG amino acid substitution model with gamma-distributed rate variation across sites (with four rate categories). An unconstrained exponential prior probability distribution on branch lengths and an exponential prior for the gamma shape parameter for among-site rate variation was applied. The final topology was estimated using 1,000,000 cycles for the metropolis-coupled Markov chain Monte Carlo analysis. The chain-heating temperature was set to 0.2. Markov chains were sampled every 200 cycles. Clade support values were calculated with posterior probabilities in MrBayes.

### In situ hybridization, nuclei staining, data documentation and developmental staging

Whole-mount in situ hybridizations were performed using a standard protocol that works for all investigated species (Additional file 4: Text file 1). Cell nuclei staining and data documentation were performed as per Janssen and Budd [23]. Determination of developmental stages follows Janssen et al. [26] (for *Glomeris*), Janssen and Budd [23] (for *Euperipatoides*), and Strobl and Stelzer [27] (for *Tribolium*).

## Results

### Gene complement and orthology

**Fig. 1:**
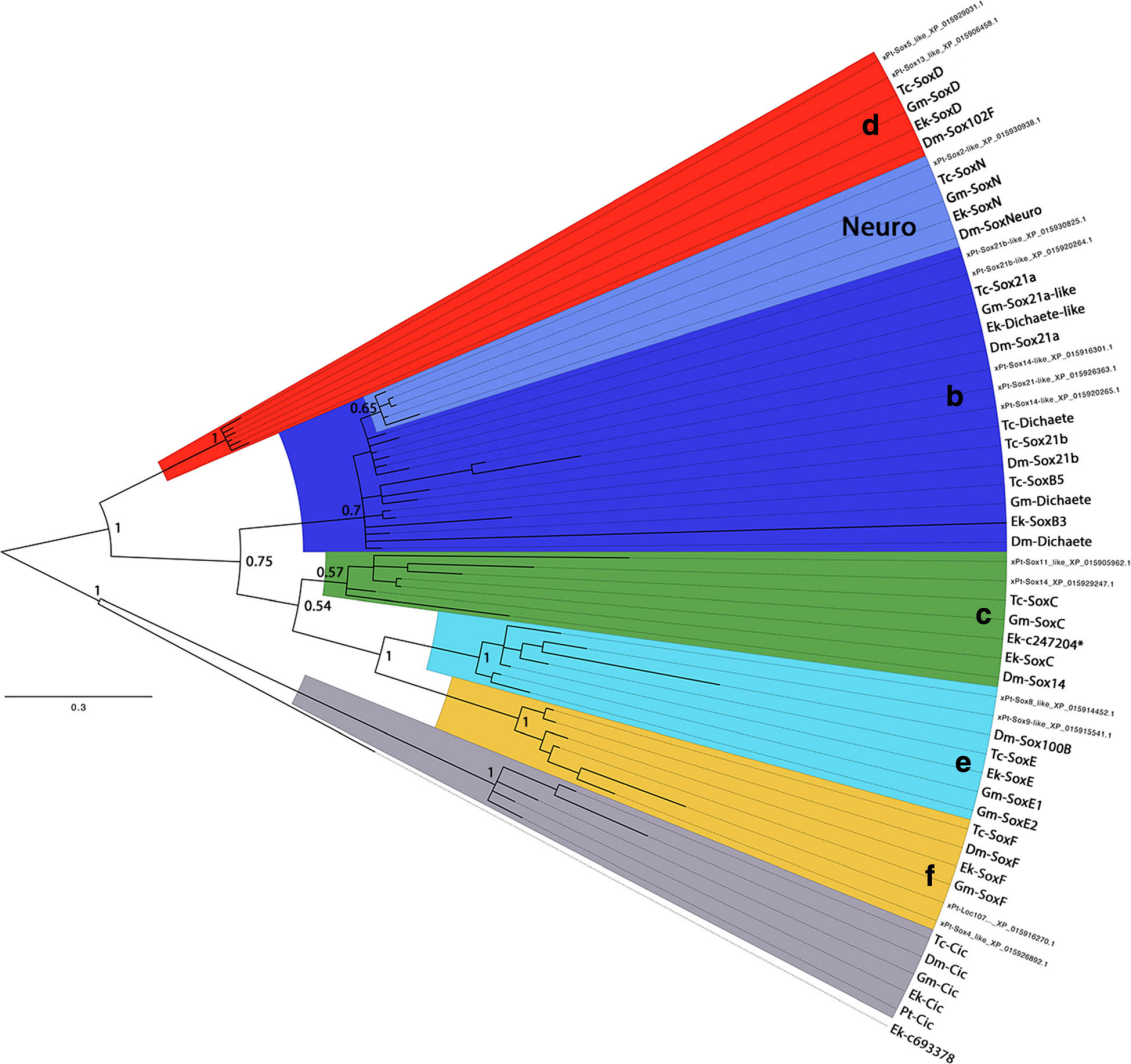
Phylogenetic analysis of Sox genes. Species abbreviations: Ek, *Euperipatoides kanangrensis* (Onychophora); Dm, *Drosophila melanogaster* (Hexapoda: Diptera); Gm, *Glomeris marginata* (Myriapoda: Diplopoda); Pt, *Parasteatoda tepidariorum* (Chelicerata: Araneae); Tc, *Tribolium castaneum* (Hexapoda: Coleoptera). Blue shades indicate Sox B class genes. Note the high support for SoxN orthologs (lighter shade of blue). Green shade: Sox C class orthologs. Red shade: Sox D class orthologs. Cyan shade: Sox E class orthologs. Yellow shade: Sox F class orthologs. As outgroups serve panarthropod Capicua (Cic) sequences and a related non-Sox/non-Cic gene sequence from the onychophoran *Euperipatoides*. Node support is given as posterior probabilities. See text for further information

Altogether, 24 Sox genes - 7 in *Euperipatoides*, 8 in *Glomeris*, and 9 in *Tribolium* - were identified. For all species, at least one ortholog that belongs to each of the five expected (based on previous studies, e.g. Wilson and Dearden [13], Cremazy et al. [10], Phochanukul and Russell [28], Paese et al. [17]) classes of arthropod Sox genes, Sox B, Sox C, Sox D, Sox E and Sox F was found. Additionally, each of the panarthropods investigated herein possess a single Sox B-class ortholog that confidently clusters with *Drosophila SoxNeuro* (*SoxN*) (Fig. 1). Overall, support for the Sox B class as a whole is relatively weak. However, since support for all other groups is high, these genes may be called Sox B genes with confidence, but their internal relationship remains unresolved (with the exception of the *SoxNeuro* orthologs). The other Sox B class genes (2 in *Glomeris*, 4 in *Tribolium*, and 2 in *Euperipatoides*) do not fall into distinct orthology groups with any sufficient support. However, conserved expression patterns (described below), have been used to define orthology groups among those genes (see Discussion).

Sox genes have been identified previously in *Glomeris marginata*, but only one of them, *SoxNeuro* has been investigated by means of whole-mount in situ hybridization (Pioro and Stollewerk [16]). Their phylogenetic analysis differs from the present analysis, suggesting a somewhat different distribution of *Glomeris* Sox genes (cf. Pioro and Stollewerk [16], their Fig. 3). In addition to their data (which were based on PCR screens with degenerate primers), a Sox D gene, and an additional Sox E gene were found. The new analysis shows further that their *SoxE1* indeed represents a Sox F gene and that their *SoxB3* is likely a PCR artifact (a hybrid of *SoxB1* and *SoxB2*). As a result, *Glomeris SoxE1* and *SoxE2* as described in this study represent *SoxE2* and *SoxE3* from Pioro and Stollewerk [16] respectively.

In this paper, we follow the nomenclature as suggested in McKimmie et al. [11] (see Table 1).

**Table 1:** Summary of commonly used synonyms for arthropod Sox genes, and gene names as used in this paper (after McKimmie et al. [5])

Species	*Drosophila* ^*b*^	*Apis*	*Tribolium*	*Glomeris*	*Euperipatoides*	*Drosophila* synonyms
	*SoxNeuro (SoxN)*	*SoxB1*	*SoxNeuro*	*SoxNeuro*	*SoxNeuro*	*SoxB1, CG18024, Sox29F*
	*Dichaete*^*a*^ (*D*)		*Dichaete* ^*c*^	*Dichaete*	*Dichaete-like*	*SoxB2–1, Fish-Hook, CG5893*
	*Sox21b* ^*a*^	*Sox21b*	*Sox21b*	*Sox21b*		*SoxB2–2, CG6419*
	*Sox21a*	*SoxB2*	*Sox21a*	*Sox21a-like*		*SoxB2–3, CG7345*
	*SoxC*	*SoxC*	*SoxC*	*SoxC*	*SoxC*	*Sox14, CG3090, Sox60B*
	*SoxD*	*SoxD*	*SoxD*	*SoxD*	*SoxD*	*Sox102F, CG11153*
	*SoxE*	*SoxE1, SoxE2*	*SoxE*	*SoxE1, SoxE2*	*SoxE*	*Sox100B, CG12098*
	*SoxF*	*SoxF*	*SoxF*	*SoxF*	*SxoF*	*Sox15, Sox50E, CG8404*
**Orphans**			*SoxB5* ^*d*^		*SoxB3* ^*d*^	

^a^Note that *Dichaete* and *Sox21b* genes have very similar expression patterns and that these genes do not resolve in phylogenetic analysis; they are therefore indistinguishable

^b^following McKimmie et al. [11]

^c^as named by Oberhofer et al. [14]

^d^as these genes cluster with Sox B class genes, but do not resolve; no expression pattern was obtained that could have used to orthologize these genes

### Gene expression patterns

#### SoxNeuro (SoxN)

**Fig. 2:**
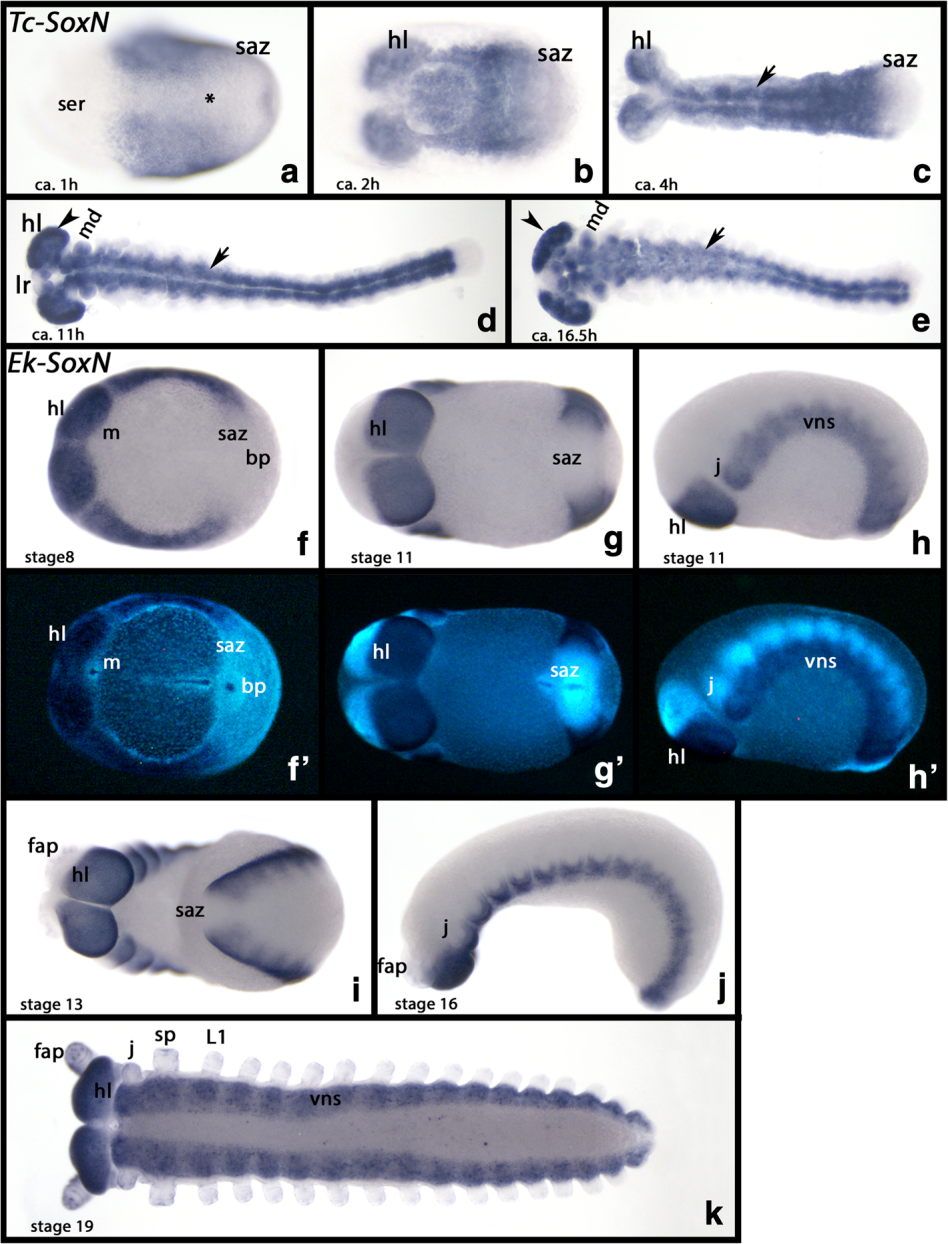
Expression of *Tribolium* and *Euperipatoides SoxNeuro* (*SoxN)*. In all panels, anterior is to the left. All panels, ventral views, except for panels **h** and **j** (lateral views). **a**-**e** Expression of *Tribolium SoxN*. **f**-**k** Expression of *Euperipatoides SoxN*. Panels **f´** to **h´** show DAPI stained embryos as shown in **f** to **h**. Developmental stages are indicated for *Tribolium* (after Strobl and Stelzer [27]) and *Euperipatoides* (after Janssen and Budd [23]). Asterisk (*) in panel **a** marks ventral tissue that expresses *SoxN* only weakly. Arrows in panels **c-e** point to expression in the VNS. The arrows in panels **d** and **e** point to expression in the brain. Abbreviations: bp, blastopore; fap, frontal appendage; j, jaw; L1, first walking limb; lr, labrum; m, mouth; md, mandible; hl, head lobe; ser, serosa; saz, segment addition zone; sp, slime papilla

*Tribolium SoxN* (*Tc-SoxN*) is expressed in the early embryo. At gastrulation, the complete embryo (except for the anterior cap that represents extraembryonic tissue) expresses *Tc-SoxN*. Shortly after, expression disappears from the future ventral midline and the posterior region of the embryo (Figs 2a and Additional file 5: Figure S2A). With the development of the embryonic germ band it becomes clear that expression of *Tc-SoxN* is strongest in the developing nervous system (Figure 2b-e). The posterior of the embryo, the segment addition zone (SAZ), remains free from expression at all developmental stages (Fig. 2). Throughout development, expression in the brain and the ventral nervous system is very strong, and it appears that all future nervous system expresses *SoxN* (Fig. 2).

The expression pattern of *Glomeris SoxNeuro* (*Gm-SoxN*) has been described previously (Pioro and Stollewerk [16]). It is strongly expressed in the brain and the ventral nerve cord, as well as in the tips of the head appendages, but not the SAZ (Pioro and Stollewerk [16]).

*Euperipatoides SoxNeuro* (*Ek-SoxN*) is expressed in the brain (the ventral half of the head lobes) and the putative ventral nervous system (all tissue ventral to the basis of the limbs) (Fig. 2f-k). As with *Glomeris* and *Tribolium*, the SAZ of *Euperipatoides* does not express *SoxN*.

#### Dichaete

**Fig. 3:**
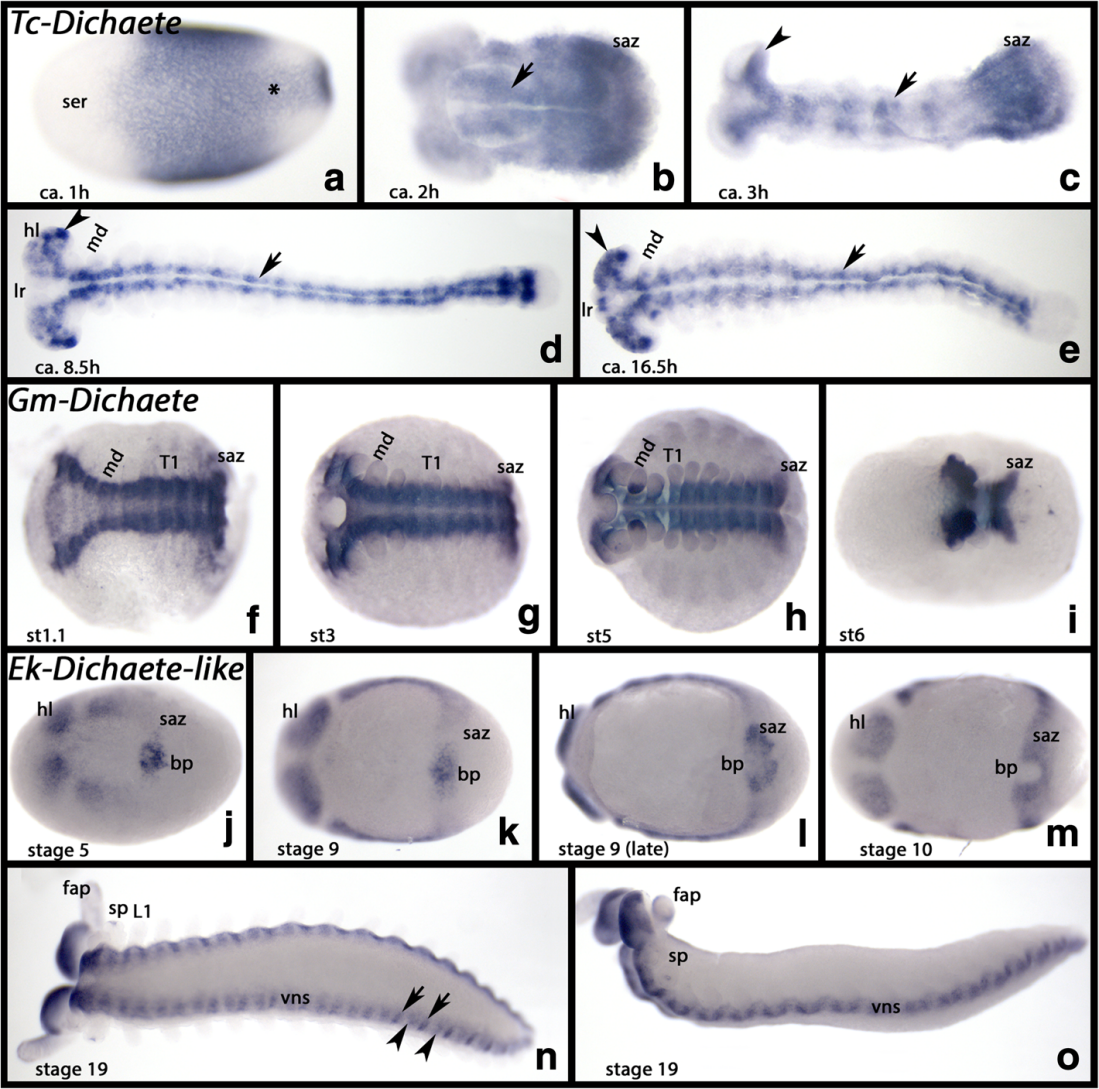
Expression of *Tribolium*, *Glomeris* and *Euperipatoides Dichaete*. In all panels, anterior is to the left. All panels, ventral views, except for panel **o** (lateral view). **a**-**e** Expression of *Tribolium Dichaete*. **f**-**i** Expression of *Glomeris Dichaete*; **j**-**o** Expression of *Euperipatoides Dichaete-like*. Developmental stages are indicated for *Tribolium* (after Strobl and Stelzer [27]), *Glomeris* (after Janssen et al. [26] ), and *Euperipatoides* (after Janssen and Budd [23]). Asterisk in panel **a** marks ventral expression (cf. expression of *SoxN*, Fig. 2a). Arrows in panels **b**-**e**, and arrowheads in panels **c**-**e** as in Fig. 2. Arrows and arrowheads in panel **n** point to different patterns of expression in the VNS. Abbreviations as in Fig. 2, and T1, first trunk segment

Shortly after gastrulation, *Tc-Dichaete* (Oberhofer et al. [14]) is expressed ubiquitously in the posterior two thirds of the developing embryo, but not the anterior cap that represents extraembryonic tissue (Additional file 5: Figure S2B). Slightly later, strong expression appears at the posterior pole of the embryo (Fig. 3a). At the same time transcripts disappear from the rest of the posterior embryo, except for a ventral stripe of expression (Fig. 3a). At later developmental stages, this ventral stripe develops into two ventral stripes that are interrupted by the ventral midline. This tissue likely represents the developing nervous system including the brain and the ventral nerve cord (Fig. 3d-e). Expression remains strong in the SAZ (Fig. 3b-d) until the end of segment addition and the beginning of germ band retraction (Fig. 3e). At these later stages, expression in the nervous system is strong, and around 16.5 h after gastrulation, additional expression appears in the labrum. Also, note the specific expression of *Tc-Dichaete* in the mandibles (Fig. 3e).

*Glomeris Dichaete* is expressed like *SoxN* with the exception that expression in the appendages is restricted to ventral dots in the mandible that first appear around stage 5. Also, unlike *SoxN*, *Gm-Dichaete* is expressed in the SAZ (Fig. 3f-i).

*Euperipatoides Dichaete-like* is expressed in similar patterns as *Ek-SoxN* with the exception that the overall expression appears less uniform and more like a salt-and-pepper pattern along the ventral nerve cord. *Ek-Dichaete-like* is also expressed in dynamic patterns in and around the posterior pit (=blastopore) and the SAZ (Fig. 3j-o).

#### Sox21b

**Fig. 4:**
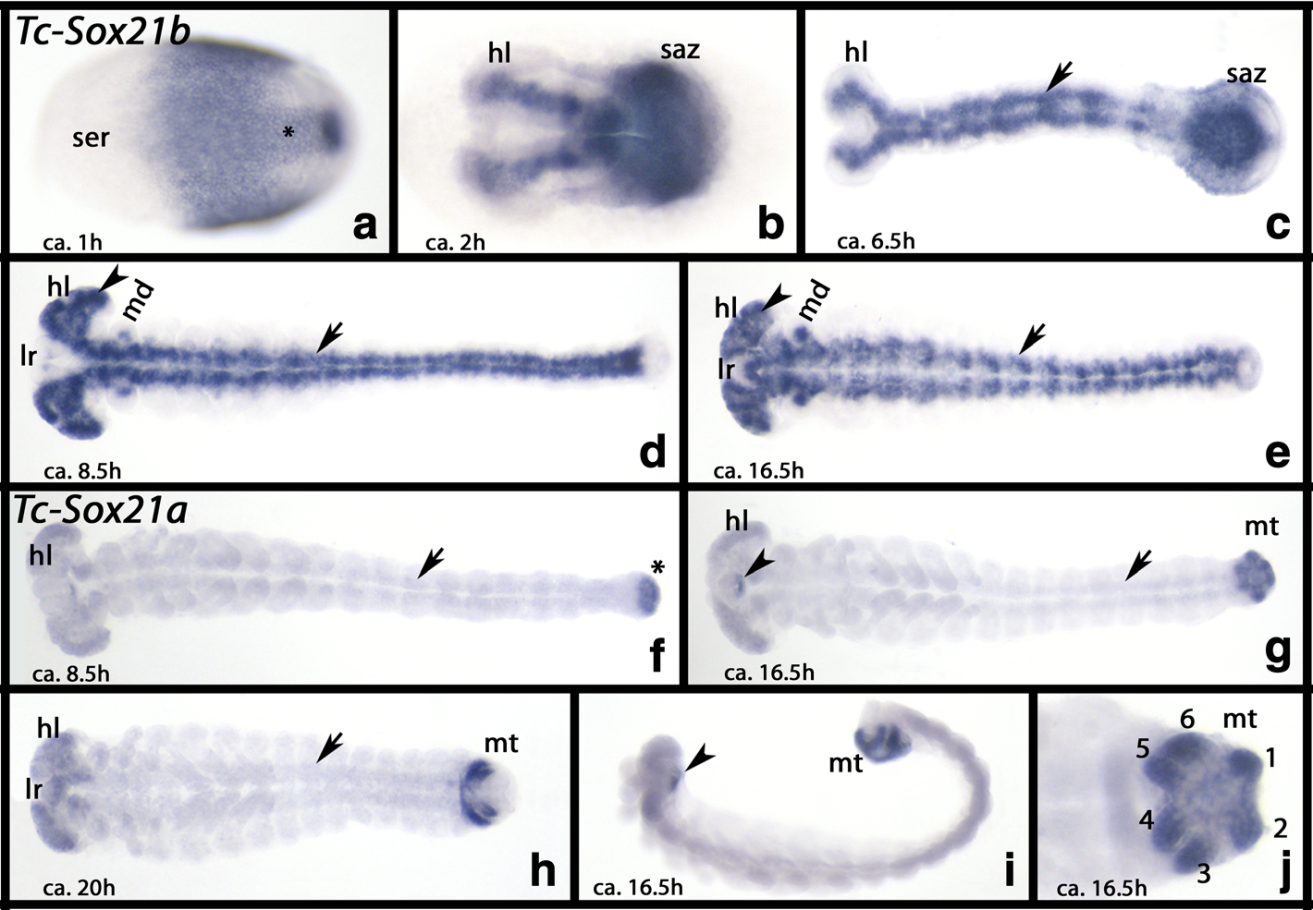
Expression of *Tribolium Sox21b* and *Sox21a*. In all panels, anterior is to the left. All panels, ventral views, except for panel **i** (lateral view). **a**-**e** Expression of *Sox21b*. **f**-**j** Expression of *Sox21a*. Developmental stages are indicated (after Strobl and Stelzer [27]). Arrows in panels **d** and **e** point to expression in the VNS (note the faint expression for *Sox21a*). Arrow in panel **i** point to expression in the mouth. Arrowheads point to expression in the head. Numbers in panel **j** mark the primordia of the Malpighian tubules. Abbreviations as in Fig. 2, and mt, Malpighian tubules

The expression of *Tribolium Sox21b* is very similar to that of *Tc-Dichaete*. Expression is uniform in the early embryo, but there is no expression in the anterior cap (Fig. 4a and Additional file 5: Figure S2C). Shortly after, dorsal posterior tissue does not express *Tc-Sox21b* anymore, and expression at the posterior pole becomes stronger (Fig. 4a). When the germ band forms and elongates, it becomes clear that expression is in the developing brain and the ventral nervous system (VNS) (Fig. 4b-e). As described for *Tc-Dichaete*, the SAZ expresses *Tc-Sox21b* strongly until all segments are formed (Fig. 4b-d). As with *Tc-Dichaete*, *Sox21b* is also specifically expressed in the mandibles, and at later stages also in the labrum, but not any other type of appendage (Fig. 4d, e).

#### Sox21a

*Tribolium Sox21a* is expressed in the primordia of the Malpighian tubules that start developing from the posterior most region of the embryo around 6.5 h after gastrulation (Fig. 4f) (cf. King and Denholm [29]). Apart from that, *Sox21a* is only expressed in the posterior half of the stomodaeal area (Fig. 4g, i), the brain (Fig. 4f-h), and weakly along the ventral midline in cells of the presumptive VNS (Fig. 4f-h). It was not possible to detect any specific expression for the short fragment of *Glomeris Sox21a-like*.

#### Other sox B class genes

No specific expression pattern for the derived *Euperipatoides SoxB3* ortholog or the *Tribolium SoxB5* ortholog was detectable in the investigated developmental stages.

#### SoxC

**Fig. 5:**
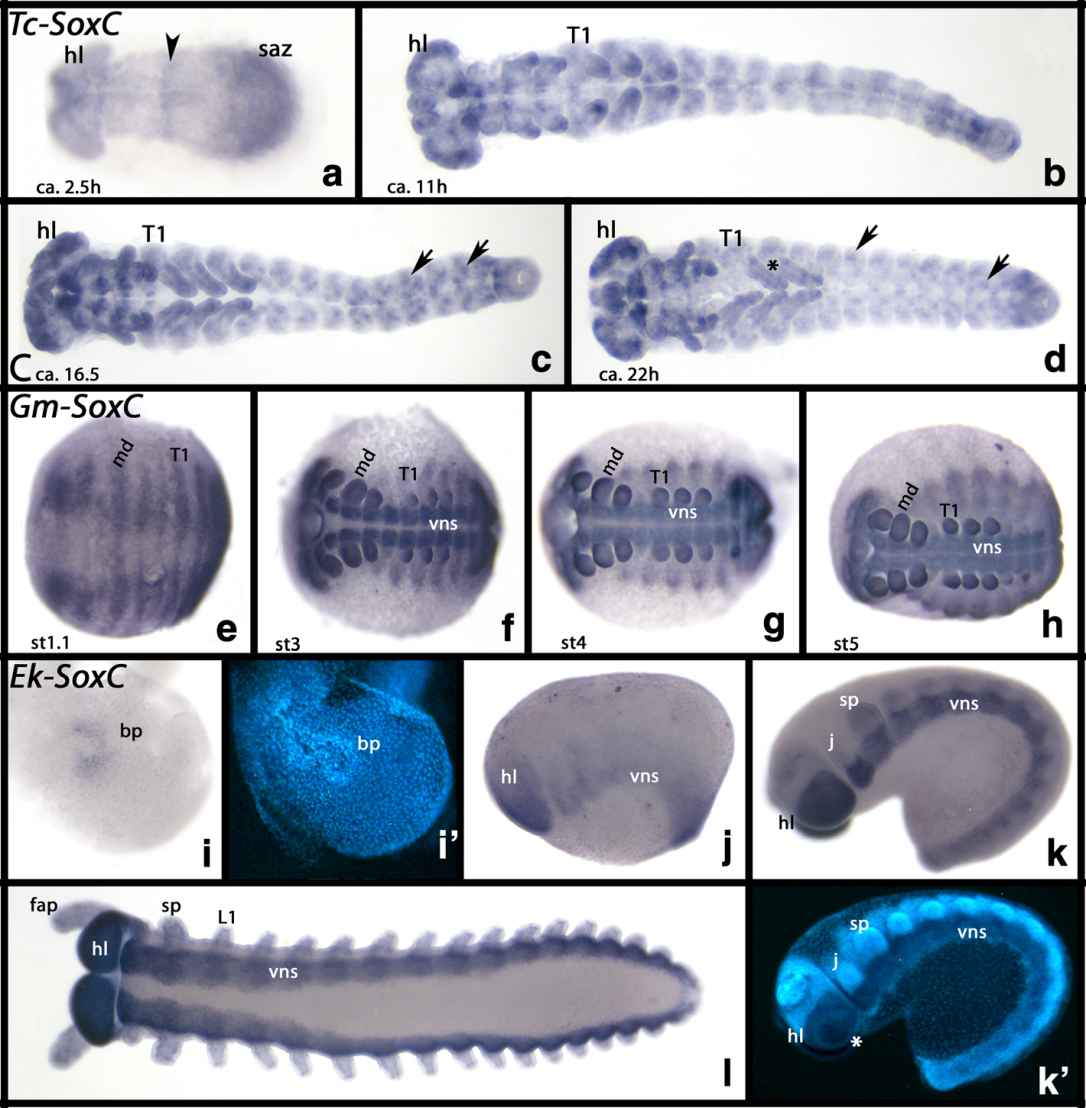
Expression of *Tribolium*, *Glomeris* and *Euperipatoides SoxC*. In all panels, anterior is to the left. All panels, ventral views, except for panels **j** and **k** (lateral views). **a**-**d** Expression of *Tribolium SoxC*. **e**-**h** Expression of *Glomeris SoxC*. **i**-**k** Expression of *Euperipatoides SoxC*. Panels **i**´ and **k**´ show DAPI stained embryos as shown in **i** and **k**. Developmental stages are indicated for *Tribolium* (after Strobl and Stelzer [27]), *Glomeris* (after Janssen et al. [26]), and *Euperipatoides* (after Janssen and Budd [23]). Arrowhead in panel a points to a single transverse stripe of expression. Arrows in panels **c** and **d** point to dot-like expression in the ventral and peripheral nervous system. The asterisk in panel **d** marks rings of expression in the walking legs. The asterisk in panel **k**´ marks rings of expression in the brain (cf. Additional file 7: Figure S3). Abbreviations as in Fig. 2, and T1, first trunk segment

*Tribolium SoxC* is expressed ubiquitously at early stages, but expression is enhanced in the SAZ and the anterior pole of the embryo (Fig. 5a). When the germ band begins to elongate, a single transverse stripe of expression appears in the middle of the embryo (Fig. 5a), and somewhat later, additional segmental stripes appear transiently (not shown).

When the limbs begin to develop, *SoxC* is expressed in their tips (Fig. 5b), and expression starts in the brain (head lobes) and in single cells along the ventral midline (Fig. 5b-d). This expression is likely associated with the formation of cells in the central as well as the peripheral nervous system. At later stages, expression is strong in the brain and in head appendages (Fig. 5b-d), faint rings of expression also appear in the walking limbs (Fig. 5d).

*Glomeris SoxC* is expressed ubiquitously (Fig. 5e). In later stages, there is stronger expression in the brain and in the ventral nervous system, similar to the expression *of SoxN* and *D* (Fig. 5f-h). At stage 5, faint transverse stripes of expression appear where the tergite borders will form (cf. expression of tergite-boundary genes in Janssen et al. [26, 30]) (Fig. 5h).

The expression pattern of *Euperipatoides SoxC* is identical with that of *SoxN* although it is stronger in the brain and the neuroectoderm ventral to the base of the jaws (Fig. 5i-l). At late developmental stages, single cells in dorsal tissue start expressing *SoxC* (not shown). Overall, there is faint ubiquitous expression (Fig. 5i-l).

#### SoxD

**Fig. 6:**
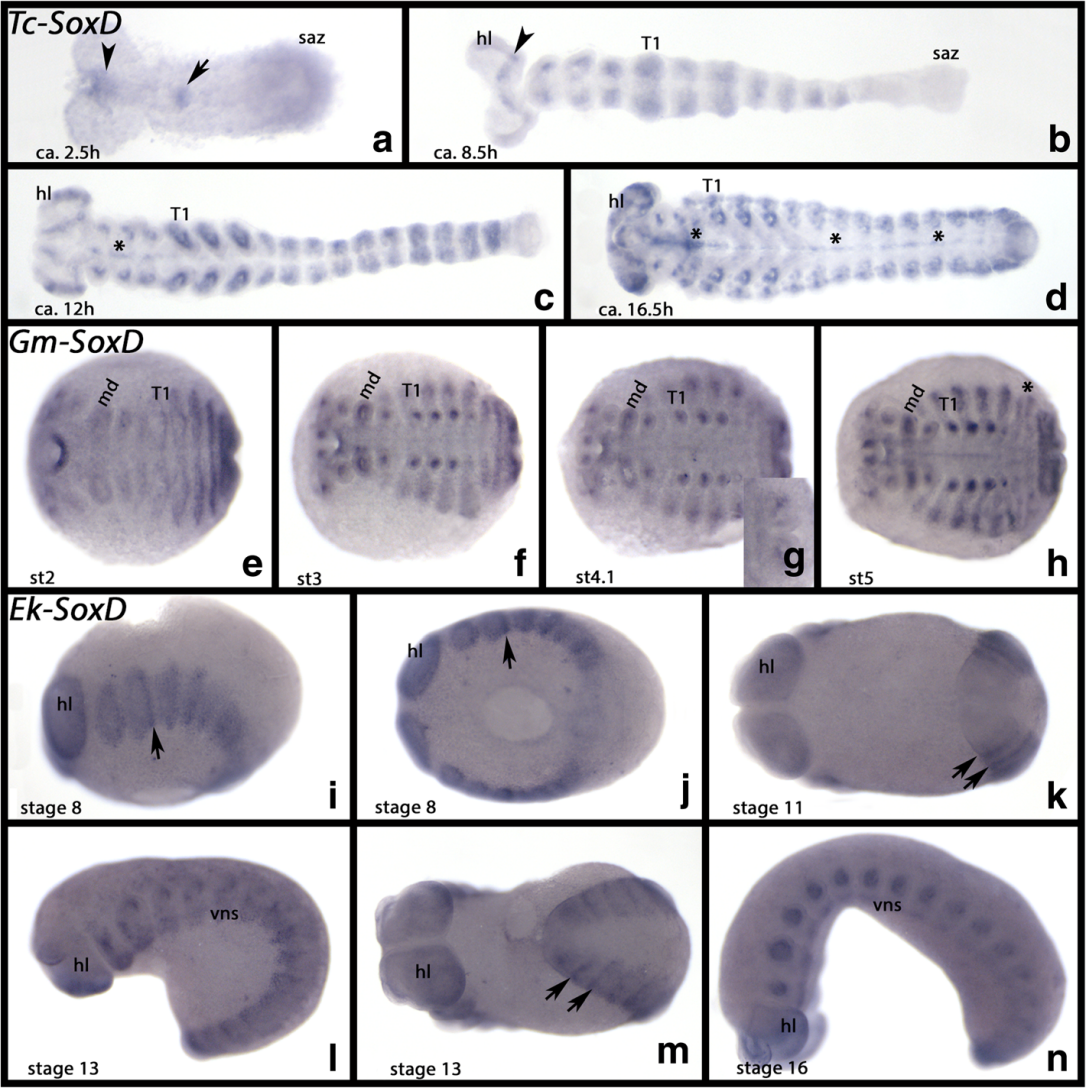
Expression of *Tribolium*, *Glomeris* and *Euperipatoides SoxD*. In all panels, anterior is to the left. All panels, ventral views, except for panels **l** and **n** (lateral views). **a-d** Expression of *Tribolium SoxD*. **e-h** Expression of *Glomeris SoxD*. **i-n** Expression of *Euperipatoides SoxD*. Developmental stages are indicated for *Tribolium* (after Strobl and Stelzer [27]), *Glomeris* (after Janssen et al. [26]), and *Euperipatoides* (after Janssen and Budd [23]). Arrowheads in panels **a** and **b** point to expression in the anterior head. Asterisks in panels **c** and **d** mark expression in the ventral midline. Asterisk in panel **h** mark two domains of expression in a diplosegmental dorsal unit (cf. Janssen [32]). The inlay in panel **g** shows expression in the SAZ and the anal valves. Arrows in panels **i** to **m** mark transverse segmental stripes of expression. Abbreviations as in Fig. 2, and T1, first trunk segment

*Tribolium SoxD* is first expressed as a single distinct dot in the future mouth region and a single ventral dot in the middle of the embryo (Fig. 6a). During germ band elongation, transverse segmental stripes appear in maturing (more anterior) segments (Fig. 6b). When the limb buds form and grow out, it becomes clear that this expression is internal and likely represents mesodermal tissue in the limbs (Fig. 6c, d). There is also expression in the ventral midline, the brain and, in stages after the beginning of germ band retraction, also along the dorsal margin of the trunk (Fig. 6c, d). This latter tissue likely contributes to the developing dorsal tube (heart), another mesodermal derivate (cf. Janssen and Damen [31]).

*Glomeris SoxD* is expressed inside (likely in mesodermal tissue) all appendages including the labrum, in the form of two spots in the brain, in the dorsal segmental units, in the posterior region of the SAZ and inside the anal valves. A weak signal is detectable in all tissue (which may represent weak expression or background) (Fig. 6e-h). Like in *Tribolium*, there is expression of *SoxD* in the ventral midline at late developmental stages (Fig. 6h). Expression in the dorsal segmental units is likely mesodermal as it is very similar to the expression of the mesodermal gene *nautilus* (*nau*) (cf. Janssen [32]).

Expression of *Euperipatoides SoxD* is complex (Fig. 6i-n). A faint ring is detectable in the brain (ventral region of the head lobes) (Fig. 6i, j). There is also expression in the ventral nervous system (ventral to the base of the limbs), but here the pattern is in a salt-and-pepper pattern (Fig. 6l, n). The SAZ is free from expression (Fig. 6j, k, m). In newly formed posterior segments, *Ek-SoxD* is expressed in a segment-polarity gene-like pattern in the form of transverse stripes at the interface of the somites/segments (Fig. 6i-k, m). *Ek-SoxD* is expressed inside (likely mesodermal) the jaws, the slime papillae and the walking limbs (Fig. 6l, n).

#### SoxE

**Fig. 7:**
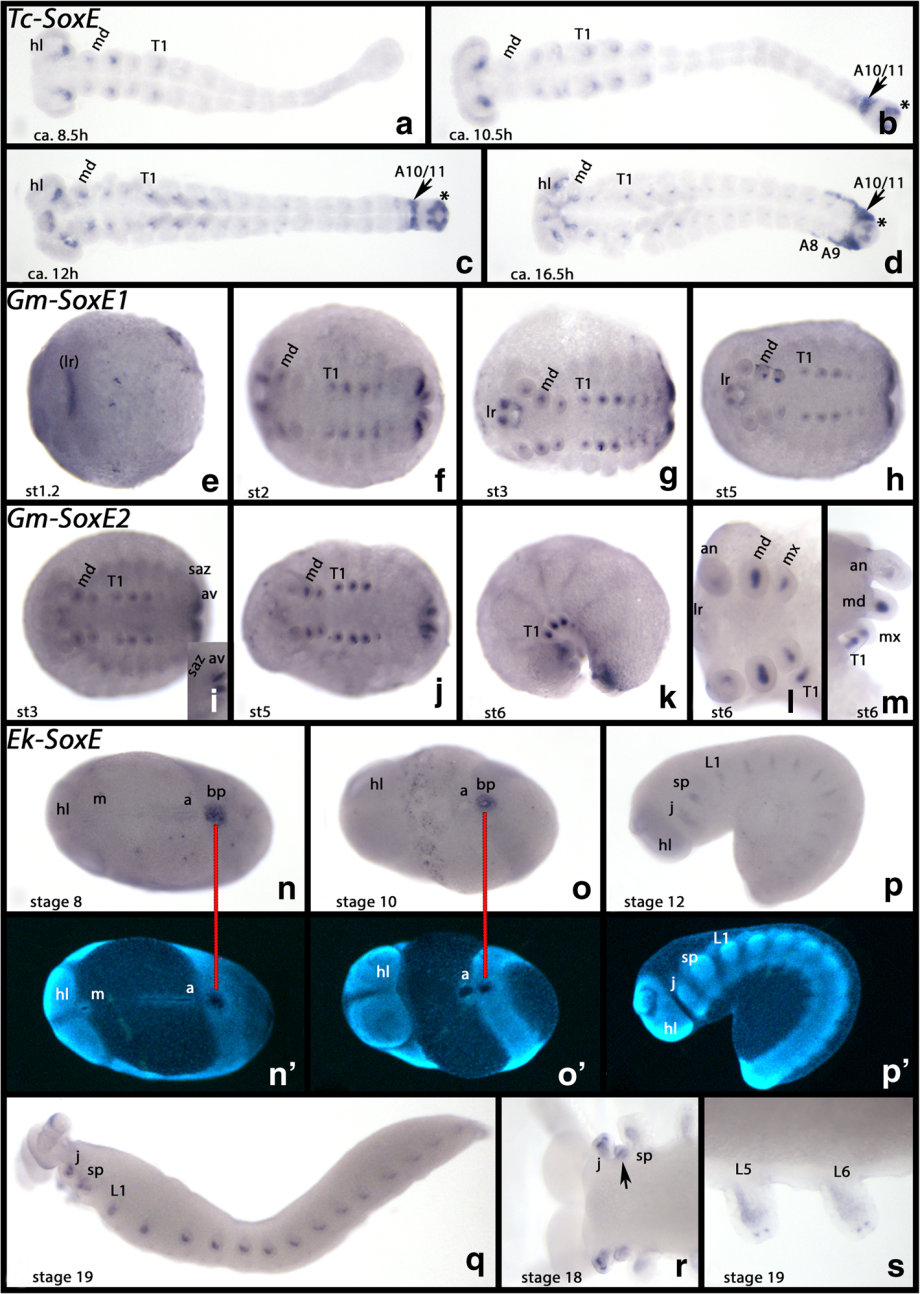
Expression of *Tribolium*, *Glomeris* and *Euperipatoides SoxE*. In all panels, anterior is to the left. All panels, ventral views, except for panels **k**, **m**, **p** and **q** (lateral views). **a**-**d** Expression of *Tribolium SoxE*. **e**-**h** Expression of *Glomeris SoxE1*. **i**-**m** Expression of *Glomeris SoxE2*. **n**-**s** Expression of *Euperipatoides SoxE*. Panels **n**´ to **p**´ show DAPI stained embryos as shown in **n** to **p**. Developmental stages are indicated for *Tribolium* (after Strobl and Stelzer [27]), *Glomeris* (after Janssen et al. [26]), and *Euperipatoides* (after Janssen and Budd [23] ). Arrows in panels **b-d** point to posterior expression, possibly associate with the formation of the Malpighian tubules. Asterisks in panels **b-d** mark the primordia of the Malpighian tubules. The inlay in panel **i** shows expression in the anal valves. Arrow in panel **r** point to expression between the jaw and the slime papilla. Abbreviations as in Fig. 2, and (lr), primordium of the labrum; a, anus; an, antenna; av, anal valves; L5 and L6, fifth and sixth trunk segment; mx, maxilla; T1, first trunk segment

*Tribolium SoxE* is not expressed at early developmental stages, but ca. 8.5 h after gastrulation expression appears inside the limb buds (Fig. 7a). It is likely that this expression represents mesodermal tissue. Approximately 10.5 h after gastrulation, additional expression appears in the form of a few dots in the very posterior of the embryo, posterior to the SAZ, and in the form of a broad domain in the now forming 10th abdominal segment (Fig. 7b). Later, the same expression is visible in the 11th abdominal segment (Fig. 7b, c). Expression in the limb buds resolves into a distinct proximal domain, in the labrum, the antennae, all gnathal appendages, the walking limbs, and even the abdominal limb anlagen (Fig. 7c, d). With the beginning of germ band retraction, the very posterior domain becomes weaker, and the expression that appeared first in the 10th and 11th abdominal segment is now restricted to the most dorsal portion, and has spread into the 8th and 9th abdominal segment as well (Fig. 7d).

The expression of *Glomeris SoxE1* (Fig. 7e-h) and *Gm-SoxE2* (Fig. 7i-m) is very similar to that described for *Gm-SoxD* with the exception that there is no detectable signal in the dorsal segmental units for *Gm-SoxE1* and *Gm-SoxE2*, and no faint ubiquitous signal. Expression in the SAZ and the anal valves is also stronger than for *Gm-SoxD*.

*Euperipatoides SoxE* is expressed in the posterior pit [the putative blastopore (Janssen et al. [33], Janssen and Budd [34])] (Fig. 7n, o) and in a segmental pattern inside the developing limbs in all segments except for the frontal appendage-bearing head lobes (Fig. 7p-r). Late during limb development two terminal dots appear in each walking limb (Fig. 7s).

#### SoxF

**Fig. 8:**
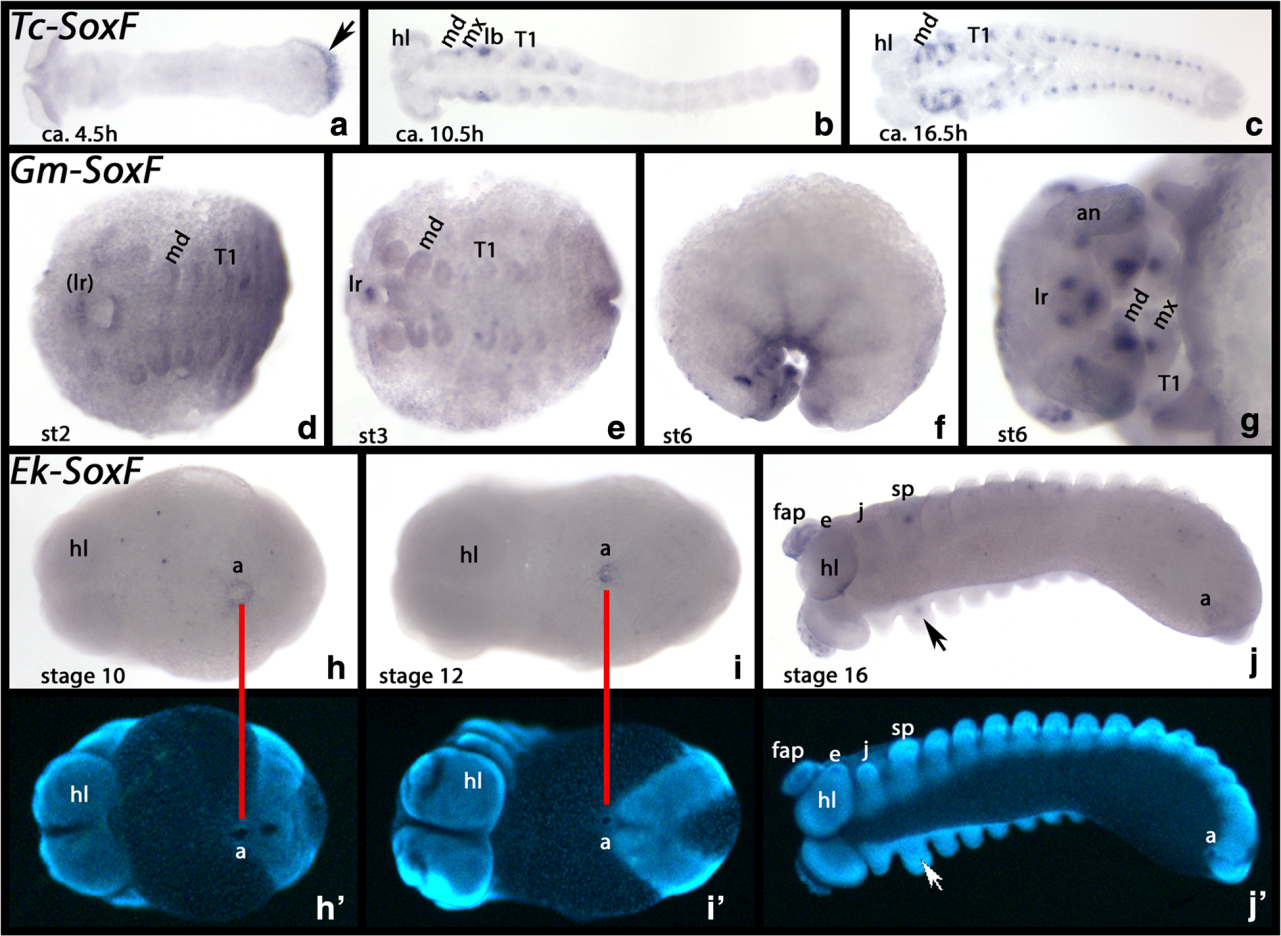
Expression of *Tribolium*, *Glomeris* and *Euperipatoides SoxF*. In all panels, anterior is to the left. All panels, ventral views, except for panel **f** (lateral views). **a**-**c** Expression of *Tribolium SoxF*. **d**-**g** Expression of *Glomeris SoxF*. **h**-**j** Expression of *Euperipatoides SoxF*. Panels **h**´ to **j**´ show DAPI stained embryos as shown in **h** to **j**. Developmental stages are indicated for *Tribolium* (after Strobl and Stelzer [27]), *Glomeris* (after Janssen et al. [26]), and *Euperipatoides* (after Janssen and Budd [23]). Arrow in panel **a** points to posterior expression. Arrow in panel **j** points to expression in the nephridia in the slime papilla Abbreviations as in Fig. 7, and e, eye; lb, labium

*Tribolium SoxF* is not expressed at early developmental stages, but with the beginning of germ band formation, expression appears at the posterior pole of the embryo. This remains the only detectable expression until approximately 4.5 h after gastrulation (Fig. 8a). At later stages, this posterior domain is not detectable any more, but de novo expression appears at the dorsal margin of the gnathal segments and in the developing walking limbs (Fig. 8b). Weak expression may also be present in the dorsal region of the head lobes (Fig. 8b). This remains the only expression until the beginning of germ band retraction. Then, additional expression appears in what seems to be the anlagen of abdominal appendages, and the expression in the walking limbs refines into a distal and a ventral and proximal dot (Fig. 8c). Similar dot-like expression is also visible in the gnathal appendages, but not the antennae or the labrum (Fig. 8c).

*Glomeris SoxF* is not expressed at early developmental stages, but at stage 2 faint expression appears ventrally in the labrum (Fig. 8d, e). Somewhat later, expression appears in the tips of the antennae, the mandibles, the maxillae and the legs (Fig. 8f, g). Additional domains of expression appear in the labrum, ventrally at the base of the antennae, and laterally in the head in a region likely associated with the formation of the eyes (Fig. 8g).

In early developmental stages, *Euperipatoides SoxF* is expressed in the anus (Fig. 8h, i). Later, expression appears in the nephridia of the slime-papilla bearing and walking-limb bearing segments (Fig. 8j). Note that expression in the fourth (L4) and the fifth (L5) walking-limb bearing segment is stronger compared with the other walking-limb bearing segments. This correlates with the enlarged nephridia in L4 and L5 (Mayer [35]).

## Discussion

### The (pan)arthropod sox gene complement

All investigated (pan)arthropod species possess at least one ortholog of Sox C, Sox D, Sox E, and Sox F, as well as a clear *SoxN* ortholog (Fig. 1, Table 2). The other Sox B class genes do not resolve well in the phylogenetic analysis (Fig. 1). However, highly conserved gene expression patterns suggest that there are orthologs of *Drosophila Dichaete* in *Tribolium* (cf. Oberhofer et al. [14]), *Glomeris* and even *Euperipatoides* (named *Dichaete-like*). While the expression of *Dichaete* orthologs is very similar to that of *SoxN*, *Dichaete* is also expressed in the SAZ and, at least in mandibulate species in the developing mandibles. A very similar pattern has been described for the spider *Parasteatoda tepidariorum* (for *Pt-Sox21b-1*) (Paese et al. [17]). In situ hybridization of two other Sox21b paralogs, however, did not reveal any embryonic expression pattern (Paese et al. [17]). The similarities in expression reflect the suggested evolutionary origin of *Dichaete*, deriving from an ancestral Sox B gene which, as a result of a duplication event, lead to *SoxN* and *Dichaete* (McKimmie et al. [11]). *Dichaete* then likely underwent another duplication event leading to *Dichaete* and *Sox21a*, and the latter likely duplicated into *Sox21a* and *Sox21b* (McKimmie et al. [11]). In *Tribolium*, one Sox B class gene is expressed in a very similar pattern as *Dichaete*. This gene clusters with *Apis Sox21b* in the analysis performed by Wilson and Dearden [13], and also shares a conserved gene expression pattern with this gene in *Apis* and *Drosophila* (Cremazy et al. [10], McKimmie et al. [11], Wilson and Dearden [13]). This gene is therefore called *Tribolium Sox21b* in this analysis.

**Table 2:**
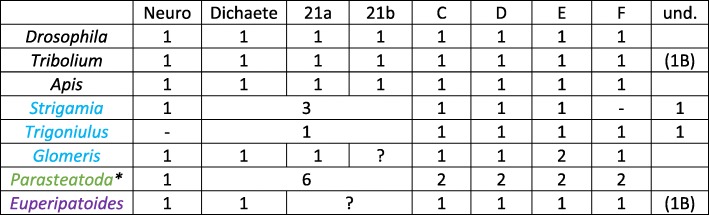
Sox gene overview

Black species names: Insecta; Blue species names: Myriapoda; Green species name: Chelicerata; Purple species name: Onychophora. Question marks indicate the unclear situation due to the lack of genome data. Data on *Trigoniulus* and *Strigamia* based on Kenny et al. [36] and Chipman et al. [101] ; Data on *Apis* based on Wilson and Dearden [13] ; Data on *Drosophila* based on Cremazy et al. [10] and McKimmie et al. [5]). Data on *Parasteatoda* are based on the sequenced genome (Schwager et al. [37] and our phylogenetic analysis). Note (*) that a whole genome duplication occurred in the lineage leading to Arachnopulmonata. Data on *Glomeris* and *Euperipatoides*: this study. Note that the data on *Glomeris* and *Euperipatoides* are based on sequenced embryonic transcriptomes, not genome data. Note that genome sequencing/annotation in *Trigoniulus* is likely not complete given the unlikely absence of some key developmental genes (Kenny et al. [36]). Abbreviations: (1B), one Class B gene without detectable expression; und., undefined

*Tribolium SoxB2* (*Sox21a* in this analysis) and *Apis SoxB2* as it is called in Wilson and Dearden [13] cluster in their analysis, but do not cluster with e.g. *Drosophila Sox21a* (see Table 1 for synonyms of arthropod Sox genes). However, these genes again share very similar expression patterns in the developing hindgut/Malpighian tubules and the foregut, as well as along the ventral midline in later stages (note that the latter is extremely difficult to detect in *Apis* and *Tribolium*) (McKimmie et al. [11], Wilson and Dearden [13], this analysis) (Fig. 4). They are therefore called *Sox21a* genes in this analysis (Tables 1 and 2). Likely, *Gm-Sox21a-like* represents a myriapod ortholog of *Sox21a*. It shows most sequence similarity in reciprocal BLAST searches with confirmed *Sox21a* genes, and it clusters with weak support with other *Sox21a* genes in the phylogenetic analysis (Fig. 1), but its expression pattern could not be analyzed because of restricted sequence information, and attempts to perform RACE failed. Two *Sox21a* genes have been discovered in the spider, but whole mount in-situ hybridization experiments did not reveal any embryonic expression pattern (Paese et al. [17]).

Insects, as far as we know, thus possess a complement of at least four Sox B class genes with highly conserved embryonic expression patterns, *SoxN*, *Dichaete*, *Sox21a*, and *Sox21b* (Table 2).

The situation in myriapods, however, is slightly more complex due to the lack of comparative gene expression data, and the fact that the data from *Glomeris* (this study) are based on a sequenced embryonic transcriptome, and not on genomic data. In addition, the data available from a second millipede, *Trioniulus corralinus* appear to be incomplete as a number of key developmental factors such as the Hox gene *proboscipedia* (*pb*), the pair rule gene ortholog *even-skipped* (*eve*) and indeed also *SoxN* have not been found (Kenny et al. [36]). In *Glomeris*, two Sox B class genes have been identified, of which one appears to be *Dichaete* (or *Sox21b*), and one is likely a *Sox21a* ortholog (*Sox21a-like*) (Table 2). In the centipede *Strigamia*, *SoxN* and three more Sox B genes have been identified and it is thus possible that they represent the same genes as found in insects, i.e. *Dichaete*, *Sox21a*, and *Sox21b* (Table 2). The recently sequenced spider *Parasteatoda tepidariorum* possesses *SoxN* and six more Sox B genes (Paese et al. [17]), which is unsurprising given the high number of duplicated genes in this species that resulted from a whole genome duplication in the lineage leading to Arachnopulmonata (Schwager et al. [37], their Table 3). In *Euperipatoides*, there is one likely *Dichaete* gene (named *Dichaete-like*), and a second, very derived Sox B class gene with unclear expression. The situation in the panarthropod ancestor is thus unclear with regard to Sox B class genes.

### A conserved role of Sox B, Sox C and Sox D class genes in nervous system development

Investigation of gene expression in early branching metazoans, Porifera and Ctenophora (e.g. Dunn et al. [38], Pick et al. [39], Pisani et al. [40]) suggests that the ancestral function of Sox genes likely was associated with the maintenance of stem cells and cell fate determination. In both groups, Sox B genes are also involved in the development of sensory cells that are likely involved in photoreception (Wiens et al. [41]). One ancestral function of Sox B genes in Metazoa is thus likely the determination of nerve cells (Jager et al. [42], Fortunato et al. [43], Schnitzler et al. [44]), a role that is also conserved in Cnidaria (Kelava et al. [45], Magie et al. [46], Shinzato et al. [47]). Sox B genes likely had redundant function early during bilaterian evolution controlling a highly-conserved gene regulatory program that is essential for the development of the embryonic nervous system (reviewed in e.g. Wegner and Stolt [48], Phochanukul and Russell [28], Neriec and Desplan [3]). *Drosophila* possesses four Sox B genes, and their importance in nervous system development is reflected by the fact that both *SoxN* and *Dichaete* retain redundant functions to secure the accurate regulation of the gene regulatory network that is in control of nervous system development. Later during development, *SoxN* and *Dichaete* are also involved in the differentiation of neurons (Russell et al. [49], Nambu and Nambu [50], Cremazy et al. [10], Buescher et al. [51], Overton et al. [52], Ferrero et al. [53]).

Sox C represents a second class of Sox genes that appears to have a general function in neurogenesis. In vertebrates, the Sox C class consists of three paralogs (*Sox4*, *Sox11* and *Sox12*). These genes play important roles late in embryonic and adult neurogenesis during neuronal differentiation when they act downstream of Sox B (e.g. Cheung et al. [54], Bergsland et al. [55], Dy et al. [56], Mu et al. [57], Chen et al. [58]). In sea urchins (Echinodermata), SoxC is involved in neuronal differentiation, but is also expressed in neuronal progenitors (Garner et al. [59], Wei et al. [60]). During neuronal differentiation, SoxC acts downstream of Six3 and is (at least partially) co-expressed with Delta (Dl) (Wei et al. [60]). In arthropods and onychophorans, *Six3* expression is mostly restricted to the head and it is thus unlikely that SoxC acts downstream of Six3 (Seo et al. 1999 [61], Posnien et al. [62], Steinmetz et al. [63], Eriksson et al. [64], Janssen [65], Hunnekuhl and Akam [66], Ortega-Hernandez et al. [67]). *Dl*, however, is expressed in similar patterns as *SoxC* in the ventral nervous system (VNS) of arthropods (Haenlin et al. [68], Dove and Stollewerk [69], Oda et al. [70], Mito et al. [71], Eriksson et al. [72]) and an onychophoran (Janssen and Budd [73]) suggesting that the interaction of SoxC and Dl may be a conserved feature of nervous system development in Bilateria. In annelids (Lophotrochozoa), *SoxB* and *SoxC* are both expressed in the VNS (Kerner et al. [74], Sur et al. [75]), implying that the general role of Sox C in nervous system development is likely conserved in Protostomia as a whole. It is therefore surprising to find that in both ecdysozoan model organisms, *Drosophila* and *Caenorhabditis*, SoxC appears to lack a function during neurogenesis (Sparkes et al. [76], Vidal et al. [12]).

The current study suggests that the general function of Sox B genes in Panarthropoda (compared to their known function in *Drosophila*) is conserved. Species representing all lineages of arthropods (Pancrustacea, Myriapoda, and Chelicerata (Paese et al. [17]) and the onychophoran *Euperipatoides* each retain at least one clear ortholog of *SoxN* and one likely ortholog of *Dichaete* and/or *Sox21b*, and both genes are expressed in very similar patterns, especially with respect to expression in the brain and the VNS (Figs. 2, 3, 4). This suggests that the interactions (such as the partial redundancy and fine tuning of the signaling) were already present in the stem leading to Panarthropoda. Furthermore, the present study shows that *SoxN* and *Dichaete* can serve as bona fide molecular markers for neuronal tissue in Panarthropoda (as suggested for *SoxN* in Mandibulata (Pioro and Stollewerk [16]).

The importance of Sox B genes in nervous system development is further highlighted by the presence of *Sox21b* type genes in insects (Wilson and Dearden [13], this study), which like *Dichaete*, are expressed in at least subsets of neuronal cells.

Unlike in *Drosophila* (Cremazy et al. [10]), in the panarthropod species investigated here, *SoxC* is differentially expressed in the brain and the VNS, and very much resembles the expression of *SoxN*, *Dichaete and Sox21b* (cf. Figs. 2, 3, 4 and 5). A comparable expression pattern has also been reported for the spider (Paese et al. [17]). This implies that Sox C class genes are likely involved in panarthropod nervous system development as well, and that the absence of Sox C in *Caenorhabditis* nervous system development and the ubiquitous expression in *Drosophila* are best explained as independently evolved derived characters. The observed expression of *SoxC* in *Tribolium* indicates a late function in nervous system development, but not a general function as indicated by the expression patterns observed in a myriapod, a spider and an onychophoran. It therefore appears likely that Sox C genes have lost their importance in nervous system development in a stepwise manner, and may have acquired other roles in insect ontogenesis.

Sox D genes are expressed in the differentiating ventral nervous system and the brain in the onychophoran and the spider (Paese et al. [17]) (Fig. 6), but these patterns are not conserved in the myriapod or any of the investigated pancrustaceans. This suggests that Sox D genes have lost their ancestral function in nervous system development, but have likely retained a general function in mesoderm development.

### Dichaete: A conserved factor in arthropod segmentation and a possible missing link in onychophoran segmentation

In *Drosophila*, *Dichaete* is expressed early during development, at the blastoderm stage, in patterns that are reminiscent of those of gap genes (GGs) and pair rule genes (PRGs). It has been shown that *Dichaete* is required for the correct expression of the primary PRGs in *Drosophila* (Russell et al. [49], Nambu and Nambu [50], Ma et al. [77]). Data on the function of *Dichaete* in *Tribolium* revealed that sequential segment addition and regulation of PRGs, like in *Drosophila*, is under control of a gene regulatory network (GRN) including transcription factors such as *caudal*, the primary PRG *odd-skipped*, and indeed also *Dichaete* (Clark and Peel [15]).

However, expression analysis in *Apis* did not reveal any Sox gene with a possible function during segmentation, suggesting that the function of *Dichaete* in *Drosophila* (Diptera) and *Tribolium* (Coleoptera) may have evolved after the split from *Apis* (Hymenoptera) (Wilson and Dearden [13], Peters et al. [78] (for an overview over insect phylogeny)).

Somewhat surprisingly, likely orthologs of *Dichaete* are expressed in dynamic patterns in the SAZ of a myriapod, an onychophoran, and of *Sox21b-1* in a spider (Paese et al. [17]) (Figs 3 and 4). Such dynamic transient expression in the SAZ is typical for PRGs in short germ arthropods (e.g. Damen et al. [79], Choe et al. [80], Janssen et al. [81]), and since PRGs are (at least partially) regulated by *Dichaete* in *Drosophila* and *Tribolium*, it appears likely that *Dichaete* and *Dichaete-like* Sox genes have played a similar role already in the last common ancestor of Panarthropoda. If so, then this function appears to have been lost in *Apis* (Wilson and Dearden [13]).

It is important to note that onychophoran segmentation appears to be regulated differently in some respects. The dynamic expression patterns of PRGs that regulate segment polarity gene (SPG) expression in arthropods are not conserved in onychophorans (Janssen and Budd [23]), and it remains unclear how the highly-conserved expression of the SPG orthologs in onychophorans (Janssen and Budd [23], Franke and Mayer [82]) is controlled without the function of PRGs (reviewed in Janssen [83]). The finding that *Dichaete* is involved in segmentation and segment addition in insects, and likely also in other arthropods and the onychophoran may offer an explanation to how SPGs are regulated in the absence of PRG function, i.e. by the function of a conserved GRN including the function of *Dichaete*.

### SoxE: A conserved factor of gonad development and mesoderm differentiation

In placental mammals, the *Sry* (*SoxA*) (sex-determining region on the Y-chromosome) gene, the archetype of Sox genes, initiates testis differentiation of the undifferentiated gonad. Shortly after, *Sox9* (an ortholog of *SoxE*, of which mammals possess three, *Sox8*, *Sox9* and *Sox10*), another testis-specific factor, is activated (reviewed in e.g. Kanai et al. [84], DeFalco and Capel [85], Barrionuevo and Scherer [86], She and Yang [87]). While *Sry* is specific to placental mammals, *Sox9* is present in all vertebrates (Morrish and Sinclair [88]). In *Drosophila*, the ortholog of *Sox9* is *Sox100B* (*SoxE*) (Fig. 1) that inter alia is expressed in the developing gonad (Loh and Russell [89], DeFalco et al. [90]), and is essential for testis development (Nanda et al. [6]). Similarly, *SoxE* is active in the development of testis in the honey bee *Apis* (Wilson and Dearden [13]) and the silkworm *Bombyx mori* (Wei et al. [91]).

This analysis shows that *Glomeris SoxE1* and *SoxE2* are both expressed in the SAZ in a region that likely harbors the gonadal primordia (Fig. 7). This is likely the case because the germ line marker *vasa* (e.g. Fujiwara et al. [92], Schröder [93], Green and Akam [94]) is expressed in a very similar (if not the same) region in the developing embryo (Janssen [95]) (cf. expression of *Glomeris vasa* Additional file 6: Figure S4). This is like the situation in *Drosophila*, where *Sox100B* is expressed in the gonadal mesoderm and in close proximity to Vasa-positive cells (Loh and Russell [89]). In the onychophoran *Euperipatoides, SoxE* is expressed in the posterior pit region, the putative blastopore (Manton [96], Janssen et al. [33]), and thus again in close proximity to *vasa*-expressing cells (cf. Fig. 7 and Additional file 6: Figure S4). In *Tribolium*, *vasa* is expressed at the posterior pole, but only during gastrulation and early germ band stages (Schröder [93]), and by the time *SoxE* is expressed, expression of *vasa* has already disappeared (cf. Fig. 7 and Schröder et al. [93]). It is therefore not possible to find the same correlation between the expression of *SoxE* and *vasa* as it appears to be the case for *Drosophila*, *Glomeris* and *Euperipatoides*. However, even in *Tribolium*, *SoxE* is expressed in the posterior abdominal segments and it is tempting to speculate that these *SoxE*-positive cells could be associated with the formation of the gonads as it is likely the case for the other arthropods and the onychophoran. Alternatively, this posterior expression could be associated with the formation of the Malpighian tubules (discussed below).

Apart from its function in gonad development, Sox E orthologs are also known to be involved in other aspects of mesoderm differentiation across Metazoa (e.g. Loh and Russell [89], Chimal-Monroy et al. [4], Akiyama et al. [97], McCauley et al. [5], Andrikou et al. [98], Schnitzler et al. [44], Focareta and Cole [99]). Data from arthropods other than *Drosophila*, however, are scarce, especially with respect to embryonic expression patterns.

We find that in all investigated arthropod species, including the spider (Paese et al. [17]), and the onychophoran *Euperipatoides*, Sox E genes are expressed in the mesoderm of the developing limbs including the labrum (Fig. 7). At least for holometabolous insects, it appears that Sox E genes are involved in the formation of the Malpighian tubules as we find *SoxE* in the primordia of these structures in *Drosophila* (Loh and Russel [89]) and *Tribolium* (Fig. 7.). But note that expression of *SoxE* was not reported for the Malpighian tubules in *Bombyx mori* (Wei et al. [100]) and *Apis mellifera* (Willson and Dearden [13]). Possibly in these species *Sox21a* could have taken over this function (see Fig. 4).

A recent study revealed the presence of two spider Sox E group genes, of which one is expressed in the mesoderm of the limb buds. The expression pattern of a second spider Sox E gene, however, remains unclear (Paese et al. [17]). The combined data on panarthropod Sox E group gene expression strongly indicate that Sox E genes act as conserved factors in panarthropod mesoderm differentiation.

## Conclusions

Panarthropods possess a conserved complement of Sox genes representing at least one member of each Sox gene family, Sox B, Sox C, Sox D, Sox E and Sox F. The provided comprehensive gene expression analysis suggests a high degree of evolutionary conservation (summarized in Additional file 8: Table S3), in which Sox B class genes are generally involved in neurogenesis, and in which the Sox B class gene *Dichaete* is likely involved in segmentation. The analysis also suggests that the insect class B gene *Sox21a* has a conserved specific function in the development of the Malpighian tubules. Similarly, Sox C orthologs appear to be involved in neurogenesis, although they seem to have (at least partially) lost this function in insects. Sox D genes appear to have a general function in panarthropod mesoderm differentiation, and have an ancestral role in nervous system development in Panarthropoda (which has been lost in Mandibulata). Sox E genes likely play a conserved role in gonadogenesis and mesoderm differentiation. The expression of Sox F genes is very diverse, and it is therefore impossible based on the currently available data to extrapolate the ancestral function of this class of Sox genes for either Panarthropoda or Arthropoda.

## Additional files

Additional file 1: **Table S1.** Primers (RTF 5 kb)

Additional file 2: **Table S2.** Accession numbers (DOCX 50 kb)

Additional file 3: **Figure S1.** Alignment (DOCX 110 kb)

Additional file 4: Whole-mount in-situ hybridization protocol. (DOCX 69 kb)

Additional file 5: **Figure S2.** Early expression of *Tribolium SoxN*, *Dichaete* and *Sox21b*. Anterior is to the left, ventral views. Developmental stages are indicated (after Strobl and Stelzer 2014). The asterisk (*) marks weaker expression in ventral tissue. Abbreviations: (saz) primordium of the segment addition zone; ser, serosa. (TIF 2612 kb)

Additional file 6: **Figure S4.** Expression of *Glomeris* and *Euperipatoides vasa* Panels (A-C), expression of *Glomeris vasa*; panels (D, E), expression of *Euperipatoides vasa*. In all panels, anterior is to the left, ventral views. Developmental stages are indicated. Panels (A´-E´) represent DAPI staining of the embryos shown in (A-E). Red bars mark same tissue in bright field and DAPI panels. Abbreviation: a, anus. (TIF 21878 kb)

Additional file 7: **Figure S3.** Expression of *Euperipatoides SoxC* in the brain. Anterior is to the left, ventral views. Developmental stages are indicated (after Janssen and Budd [23]). Arrows point to dynamic expression in the brain (best seen in DAPI stained embryos). Abbreviations: hl, head lobe; vns, ventral nervous system. (TIF 10243 kb)

Additional file 8: **Table S3.** Overview expression data (DOCX 91 kb)

## Abbreviations

DAPI: 4–6-Diamidino-2-phenylindole
GG: gap gene
GRN: gene regulatory network
PRG: pair rule gene
RACE: rapid amplification of cDNA ends
SAZ: segment addition zone
SPG: segment polarity gene
VNS: ventral nervous system.
