# Supplementary material for: Embryonic expression patterns and phylogenetic analysis of panarthropod sox genes: insight into nervous system development, segmentation and gonadogenesis
Source: BMC Evol Biol. 2018 Jun 8;18:88. doi: 10.1186/s12862-018-1196-z (PMC5994082; doi:10.1186/s12862-018-1196-z)
Supplement: Supplementary file 1 — Table S1. Primers (RTF 5 kb) [file 12862_2018_1196_MOESM1_ESM.rtf]

EuperipatoidesSoxN_fw1_810		TAAAAGGAGGTAGTATGGCG	SoxN_fw2_828		CGCCACAAACTAATACAACT	SoxN_bw1_2107		CATACTTACTGAAACCTGCCSoxN_bw2_2050		TACGAAAAATCATCCCTTGC	D-like_fw1_325		AAATGGCTAAACCTGCGGATD-like_fw2_364		ACGCTTTTATGGTATGGTCGD-like_bw1_1238		AGTGACCATACTTTCATTCGD-like_bw2_1224		CATTCGTAGATCCTCTTGTGSoxB3_fw1_344		ACAGCCTCATAAAGTTGACASoxB3_fw2_368		ACAACCTGGATGAATGGCTTSoxB3_bw1_1486 		TACTCTGCATAAAAGAAGCCSoxB3_bw2_1145		TCTTATCGAGACCCTAAACASoxC_fw1_922		CGTCTACACCTTATACAGATSoxC_fw2_963		TCCAGTCATATTAAACGTCCSoxC_bw1_2031		CACACTTTACTCAGATTTGGSoxC_bw2_2014		TGGTCGAGTGCATAGTTTACSoxD_fw1_910		TGGGCAGCAACAGCACAAGASoxD_fw2_931		CCAGCAACTTCAGCATCAGASoxD_bw1_2245		ATTCAGTCCATTGTCACGGTSoxD_bw2_2213		GCATCTCTTGTCGTCTCACASoxE_fw1_901		TCGTGAAGCTGTGACAAAAGSoxE_fw2_928		AGGTTATGACTGGACTTTGGSoxE_bw1_2059		GGAAGTGGAAGGAGATGAAGSoxE_bw2_2031		ATTGAAGGGAATATGGTGCCSoxF_fw1_537		GTGCTAAGGAAAATGGAGAASoxF_fw2_562		TCGCAATAGTCGTAAATCGGSoxF_bw1_1762		CGTACTATTAACAGAACTGCSoxF_bw2_1702		GCCACTGCTGTTACTGTATT—Glomeris D_fw1_476			GAACGCTTTCATGGTATGGTD_fw2_523			AGGAGAACCCGAAGATGCACD_bw1_1915			TCGGTTTCTGTGTGCAAAGAD_bw2_1692			CCTCCCCTATGCAATTAACASox21a-like_fw1_1	GTTAAAAGGAGGTAGTATGGSox21a-like_fw2_12	GTAGTATGGCGCCACAAACTSox21a-like_bw1_260	GTTAGCTTCATCTATAAAGGSox21a-like_bw2_239	GCCTTTTTTCAGACTCCGACSoxC_fw1_1870		GAGCAAAATGTTCGGTTCGCSoxC_fw2_1908		CGTCAACGCCATACACGGATSoxC_bw1_3068		GGATGAAGATGACGAACTCCSoxC_bw2_3037		TCGTTGCCACAGCGTAGAATSoxD_fw1_689		TCATGGGTACACCTTACGGASoxD_fw2_726		TATGCCCACAGCTCTTAACCSoxD_bw1_1889		GCGATACGTGCATTTCTACCSoxD_bw2_1863		CAATCTCTCCTATGGCACAGSoxE1_fw1_1			GCAACAACAGCAGCAGCAACSoxE1_fw2_12		AGCAGCAACAGCATCCTTTCSoxE1_bw1_483		TACTTGTAGTCAGGGTGTTCSoxE1_bw2_466		TTCCTTCTTGTGTATGAGTCSoxE2_fw1_277		CCATCAAATGCCACCACCACSoxE2_fw2_302		CCACCATAACCATAGGAGGASoxE2_bw1_1359		GTTTATCGAGTTGCTACCGGSoxE2_bw2_1336		CCGACGCAGATGGACGGTAASoxF_fw1_1			GCTAAAGTGGAAAGAAAGAGSoxF_fw2_22			CTTGCTGATGAAAATCCAGASoxF_bw1_1261		CTCCTCCTACAGATGCAGTTSoxF_bw2_1214		TGGTCAAACTCAGCCCTATC-TriboliumSoxN_fw1_2			TGTTGACGATGGAAACGGACSoxN_fw2_58			CACCACGCCATACAGTCCTASoxN_bw1_930		ATCGGTGGGCAGGTACATGGSoxN_bw2_771		CGTCTGTTGCATTCCGTAGGD_fw1_6				CCTTTTAACAACCGACACCC	D_fw2_43			AGCTTCGCCCAAGTTCATGTD_bw1_752			TCAATACATAACTGGGACCGD_bw2_679			GCCGAGTACATCATGGGCGASoxB21b_fw1_72		CGTCCCTCAAGGTCTGCAAA		SoxB21b_fw2_104		CGAAGAAGCAAGAAGACCACSoxB21b_bw1_856		AGGCGTCCGAGTACATCTTGSoxB21b_bw2_804		CGGGTTGTACTTGCTGTCCASoxB21a_fw1_56		ACGCCTTCATGGTATGGTCASoxB21a_fw2_116		GCCCAAGAGAACCCGAAAATSoxB21a_bw1_729		TCATATAACGTGTCCAGCGGSoxB21a_bw2_674		ATAACGCAGACGGGTCGCTTSoxB5_fw1_4			TCCGACAGTGATAACAGTGCSoxB5_fw2_24		CCACATCAAAAGACCCATGASoxB5_bw1_624		CTACAGAGGCGGAAAATACASoxB5_bw2_597		AGAGTTTCCAGCCAAATCGTSoxC_fw1_7			GCGTTTATGGTGTGGTCTCASoxC_fw2_46			TGCGAAGTGCAGCCAGACATSoxC_bw1_735		CGAGAAGATGGGGATGTCTCSoxC_bw2_709		AGTCAAAGTCCAGGTGCGAGSoxD_fw1_159		AGGCGGCACGATGGTCAATASoxD_fw2_218		CGAGTGTTTCAGGCAGCGAASoxD_bw1_1318		GCATCGGGAAATGAGGTTGCSoxD_bw2_1262		AGTGGACTAAACTGTGGGGGSoxE_fw1_69			CGTTTCGGTTCTCCAAGATASoxE_fw2_97			TCACAGACAATCAACGGTGGSoxE_bw1_1150		TGTAATGTCCCCACGGTTCASoxE_bw2_1124		CTCTCACCGTTGTAAGTCAGSoxF_fw1_13			ACTTCGTCTCCCACAACTGCSoxF_fw2_46			AATTGCTTGTCCCCGTCCAGSoxF_bw1_1182		AGCACTCATCACGTACATCCSoxF_bw2_1146		CGAAGTGATGGGTTGGATGT
