# Supplementary material for: Embryonic expression patterns and phylogenetic analysis of panarthropod sox genes: insight into nervous system development, segmentation and gonadogenesis
Source: BMC Evol Biol. 2018 Jun 8;18:88. doi: 10.1186/s12862-018-1196-z (PMC5994082; doi:10.1186/s12862-018-1196-z)
Supplement: Supplementary file 2 — Table S2. Accession numbers (DOCX 50 kb) [file 12862_2018_1196_MOESM2_ESM.docx]

| Gene | Accession no. |
| --- | --- |
| *Gm-Dichaete* | LT908049 |
| *Gm-SoxC* | LT908050 |
| *Gm-SoxD* | LT908051 |
| *Gm-SoxE1* | LT908052 |
| *Gm-SoxE2* | LT908053 |
| *Gm-SoxF* | LT908054 |
| *Ek-Neuro* | LT907951 |
| *Ek-Dichaete-like* | LT907952 |
| *Ek-SoxB3* | LT907953 |
| *Ek-SoxC* | LT907954 |
| *Ek-SoxD* | LT907955 |
| *Ek-SoxE* | LT907956 |
| *Ek-SoxF* | LT907957 |
| *Tc-Neuro* | LT908033 |
| *Tc-Dichaete* | LT908025 |
| *Tc-Sox21a* | LT908026 |
| *Tc-Sox21b* | LT908027 |
| *Tc-SoxB5* | LT908028 |
| *Tc-SoxC* | LT908029 |
| *Tc-SoxD* | LT908030 |
| *Tc-SoxE* | LT908031 |
| *Tc-SoxF* | LT908032 |
