# Supplementary material for: Embryonic expression patterns and phylogenetic analysis of panarthropod sox genes: insight into nervous system development, segmentation and gonadogenesis
Source: BMC Evol Biol. 2018 Jun 8;18:88. doi: 10.1186/s12862-018-1196-z (PMC5994082; doi:10.1186/s12862-018-1196-z)
Supplement: Supplementary file 3 — Figure S1. Alignment (DOCX 110 kb) [file 12862_2018_1196_MOESM3_ESM.docx]

Pt-Sox14-like X  1 RIRRPMNAFMVWSRAQRRKIALEHPKMHNSEISKRLGVEWKQLDENEKRPFIDEAKRLRQ 60

Tc-SoxB5         1 HIKRPMNAFMVWSRIRRKHISRDYPRLHNSEISKLLGAEWKVLPEAEKRPFIDEAKRLRN 60

Pt-Sox5 like XP  1 HIKRPMNAFMVWAKDERRKILKACPDMHNSNISKILGARWKAMTNSEKQPYYEEQSRLSR 60

Dm-Sox102F       1 HIKRPMNAFMVWAKDERRKILKACPDMHNSNISKILGARWKAMSNADKQPYYEEQSRLSK 60

Tc-SoxD          1 HIKRPMNAFMVWAKDERRKILKACPDMHNSNISKILGARWKSMSNAEKQPYYEEQSRLSK 60

Pt-Sox13 like X  1 HIKRPMNAFMVWAKDERRKILKACPDMHNSNISKILGARWKAMSNGEKQPYYEEQSRLSK 60

Gm-SoxD          1 HIKRPMNAFMVWAKDERRKILKACPDMHNSNISKILGARWKAMSNAEKQPYYEEQSRLSK 60

Ek-SoxD          1 HIKRPMNAFMVWAKDERRKILKACPDMHNSNISKILGAKWKAMSNAEKQPYYEEQSRLSK 60

Ek-SoxB3         1      MNGFMMFSRLNRRHFIKLNPGVATSQISKMLGAEWRRMNEEEQKPYREKA      50

Ek-c693378       1 HIKRPMNAFMLFQYEVRPILLKQFPDKTNKEISVLLGAKWKSLPDFEKQKYYNMAKDVAR 60

Dm-Cic           1 KIRRPMNAFMIFSKKHRKMVHKKHPNQDNRTVSKILGEWWYALKPEQKAQYHELASSVKD 60

Gm-Cic           1 HIRRPMNAFMIFSKRHRPLVHQRHPNQDNRTVSKILGEWWYALDPQQKKEYHDLAFQVKE 60

Tc-Cic           1 RIRRPMNAFMIFSKRHRALVHQRHPNQDNRTVSKILGEWWYALGPEQKRKYHELASEVKE 60

Ek-Cic           1 HIRRPMNAFMIFSKRHRALVHQRHPNQDNRTVSKILGEWWYALGPLEKQKYHDLAFQVKE 60

Pt-Cic           1 HIRRPMNAFMIFSKRHRALVHQRHPNQDNRTVSKILGEWWYALEPERKQKYHDLAYEVKE 60

Pt-Loc107... XP  1 RIRRPMNAFMVWAKSERKRLADENPDMHNADLSKLLGKRWRSLTPAERQPYVQEAERLRQ 60

Pt-Sox4 like XP  1 RIRRPMNAFMVWAKVERKRLADENPDLHNADLSKMLGKKWRGLSHDDRRPYVEEAERLRV 60

Gm-SoxF          1             AKVERKRLADENPDLHNADLSKMLGKKWRSLTPSDRRPYVEEAERLRV 48

Ek-SoxF          1 RIRRPMNAFMVWAKAERKRLADENPDLHNADLSKMLGKKWRSLTPIDRRPFVEEAERLRV 60

Tc-SoxF          1 RIRRPMNAFMVWAKVERKKLADENPDLHNADLSKMLGKKWRSLTPQDRRPFVEEAERLRV 60

Dm-Sox15         1 RIRRPMNAFMVWAKIERKKLADENPDLHNADLSKMLGKKWRSLTPQDRRPYVEEAERLRV 60

Dm-Sox100B       1 HIKRPMNAFMVWAQAARRVMSKQYPHLQNSELSKSLGKLWKNLKDSDKKPFMEFAEKLRM 60

Pt-Sox9-like XP  1 YVKRPMNAFMVWAQAARRKLADQYPHLHNAELSKTLGKLWRLLNEDDKRPFVEEAERLRI 60

Tc-SoxE          1 HVKRPMNAFMVWAQAARRKLADQYPQLHNAELSKTLGKLWRLLSDVDKKPFIEEAERLRV 60

Pt-Sox8 like XP  1 HVKRPMNAFMVWAQAARRKLADQYPHLHNAELSKTLGKLWRVLGDDEKKPFIEEAERLRC 60

Ek-SoxE          1 HVKRPMNAFMVWAQAARRKLADQYPHLHNAELSKTLGKLWRLLNDDEKKPFIEEAERLRV 60

Gm-SoxE1         1 HIKRPMNAFMVWAQAARRKLADHYPHLHNAELSKTLGKLWRLLSDDEKRPFIEEAERLRV 60

Gm-SoxE2         1 HVKRPMNAFMVWAQASRRKLSDHYPHLHNAELSKTLGKLWRLLSDDEKRPFIEEADRLRL 60

Pt-Sox11 like X  1 HVKRPMNPFMIWSQIERRKIMDQNPGIPSPEISVQLGKRWKMLTNQERIPFAKEAERLRK 60

Pt-Sox14 XP_015  1 HVKRPMNAFMVWSQIERRRICEEQPDMHNAEISKRLGMMWRLLDEDQRKPFVEEADRLRQ 60

Dm-Sox14         1 HIKRPMNAFMVWSQMERRKICERTPDLHNAEISKELGRRWQLLSKDDKQPYIIEAEKLRK 60

Tc-SoxC          1 HIKRPMNAFMVWSQIERRKICEVQPDMHNAEISKKLGRRWKELKDDERQPFIEEAERLRQ 60

Ek-SoxC          1 HIKRPMNAFMVWSQIERRKICEHHPDMHNAEISKRLGKRWKLLNDEEKQPYIEEAERLRI 60

Gm-SoxC          1 HIKRPMNAFMVWSQIERRKICEQQPDMHNAEISKRLGKRWKMLSDVERQPFIEEAERLRI 60

Ek-247204        1 HIKRPMNAFMVWSQIERRKICEQQPDMHNAEISKRLGKRWKMLSDVERQPFIEEAERLRI 60

Ek-D-like        1 HVKRPMNAFMVWSRGQRRKMAQENPKMHNSEISKRLGAEWKLLPESEKRPFIDEAKRLRA 60

Tc-SoxB2         1 HIKRPMNAFMVWSRGQRRKMAQENPKMHNSEISKRLGAEWKLLSESEKRPFIDEAKRLRA 60

Gm-Sox21a        1      MNAFMVWSRGQRRKMAQENPKMHNSEISKRLGAEWKLLSESEKRPFIDEAN     51

Dm-SoxN          1 RVKRPMNAFMVWSRGQRRKMASDNPKMHNSEISKRLGAQWKDLSESEKRPFIDEAKRLRA 60

Tc-SoxN          1 RVKRPMNAFMVWSRGQRRKMAQENPKMHNSEISKRLGAEWKLLSEAEKRPFIDEAKRLRA 60

Gm-SoxN          1 RVKRPMNAFMVWSRGQRRKMAQENPKMHNSEISKRLGAEWKLLSEAEKRPFIDEAKRLRA 60

Pt-Sox2-like XP  1 RVKRPMNAFMVWSRGQRRKMAQENPKMHNSEISKRLGAEWKLLSESDKRPFIDEAKRLRA 60

Ek-SoxN          1 RVKRPMNAFMVWSRGQRRKMAQENPKMHNSEISKRLGAEWKLLSESEKRPFIDEAKRLRA 60

Pt-Sox21b-like   1 HVKRPMNAFMVWSRGQRRKLAQENPKMHNSEISKRLGAEWKLLSEAEKRPFIDEAKRLRA 60

Pt-Sox21b-like   1 HVKRPMNAFMVWSRAQRRKMAQENPKMHNSEISKRLGAEWKLLTEPEKRPFIDEAKRLRA 60

Gm-D             1 HVKRPMNAFMVWSRAQRRKIAQENPKMHNSEISKRLGAEWKLLTESEKRPFIDEAKRLRA 60

Dm-Sox21a        1 HIKRPMNAFMVWSRGQRRKMAQDNPKMHNSEISKRLGAEWKLLTEGQKRPFIDEAKRLRA 60

Dm-D             1 HIKRPMNAFMVWSRLQRRQIAKDNPKMHNSEISKRLGAEWKLLAESEKRPFIDEAKRLRA 60

Tc-Sox21b        1 HIKRPMNAFMVWSRLQRRKIAQENPKMHNSEISKRLGAEWKLLTEDEKRPFIDEAKRLRA 60

Dm-Sox21b        1 HIKRPMNAFMVWSRLQRRKIAQDNPKMHNSEISKRLGAEWKLLTEEEKRPFIDEAKRLRA 60

Tc-D             1 HIKRPMNAFMVWSRIQRRKIALENPKMHNSEISKRLGAEWKLLSEVDKRPFIDEAKRLRA 60

Pt-Sox14-like X  1 HVKRPMNAFMVWSRAQRRKIALENPKMHNSEISKRLGTEWKHLSEQEKRPFIEEAKRLRA 60

Pt-Sox21-like X  1 RIRRPMNAFIIWSRVQRRRIARENPQLHNSEISKRLGVEWKQMNENEKRPFINEAEHLRQ 60

                                   *       *      .*  **  *  .    .  .

Pt-Sox14-like X 61 EHMRLHPDYKYRPRRKP 77

Tc-SoxB5        61 QHMVDHPDYKYRPRRKP 77

Pt-Sox5 like XP 61 LHMEKHPDYRYRPRPKR 77

Dm-Sox102F      61 LHMEQHPDYRYRPRPKR 77

Tc-SoxD         61 LHMEKHPDYRYRPRPKR 77

Pt-Sox13 like X 61 LHMEKHPDYRYRPRPKR 77

Gm-SoxD         61 LHMEKHPDYRYRPRPKR 77

Ek-SoxD         61 LHMEKHPDYRYRPRPKR 77

Ek-SoxB3        51                   50

Ek-c693378      61 RHSQMYPDYVYNPKLRR 77

Dm-Cic          61 AHFKLHPEWKWCSKDRR 77

Gm-Cic          61 AHFRAHPDWKWCSRDRK 77

Tc-Cic          61 AHFKAHPEWKWCNKDRR 77

Ek-Cic          61 AHFKAYPDWKWCSKDRK 77

Pt-Cic          61 AHFKAYPNWKWCSRDRK 77

Pt-Loc107... XP 61 QHQKDYPHYKYRPRRRK 77

Pt-Sox4 like XP 61 QHMHDYPNYKYRPRRRK 77

Gm-SoxF         49 LHMQEHPNYKYRPRRRK 65

Ek-SoxF         61 LHMQEHPNYKYRPRRRK 77

Tc-SoxF         61 IHMTEHPNYKYRPRRRK 77

Dm-Sox15        61 IHMTEHPNYKYRPRRRK 77

Dm-Sox100B      61 THKQEHPDYKYQPRRKK 77

Pt-Sox9-like XP 61 IHKREHPDYKYQPRRKK 77

Tc-SoxE         61 IHKREHPDYKYQPRRRK 77

Pt-Sox8 like XP 61 KHKKDYPDYKYQPRRRK 77

Ek-SoxE         61 VHKKEHPDYKYQPRRRK 77

Gm-SoxE1        61 IHKKEHPDYKYQPRRRK 77

Gm-SoxE2        61 IHKKEHPDYK        70

Pt-Sox11 like X 61 LHSQEFPDYKFSPKPKK 77

Pt-Sox14 XP_015 61 LHQIQYPDYKYRPRKKK 77

Dm-Sox14        61 LHMIEYPNYKYRPQKKQ 77

Tc-SoxC         61 LHQREYPDYKYRPRKKT 77

Ek-SoxC         61 LHMQEYPDYKYRPRKKV 77

Gm-SoxC         61 LHMQEYPDYKYRPRKKA 77

Ek-247204       61 LHMQEYPDYKYRPRKKA 77

Ek-D-like       61 LHMKEHPDYKYRPRRKP 77

Tc-SoxB2        61 LHMKEHPDYKYRPRRKP 77

Gm-Sox21a       52                   51

Dm-SoxN         61 VHMKEHPDYKYRPRRKT 77

Tc-SoxN         61 VHMKEHPDYKYRPRRKT 77

Gm-SoxN         61 VHMKEHPDYKYRPRRKT 77

Pt-Sox2-like XP 61 VHMKEHPDYKYRPRRKT 77

Ek-SoxN         61 VHMKEHPDYKYRPRRKT 77

Pt-Sox21b-like  61 LHMKEHPDYKYRPRRKP 77

Pt-Sox21b-like  61 VHMREHPDYKYRPRRKP 77

Gm-D            61 QHMKDHPDYKYRPRRKP 77

Dm-Sox21a       61 LHMKEHPDYKYRPRRKP 77

Dm-D            61 LHMKEHPDYKYRPRRKP 77

Tc-Sox21b       61 MHMKEHPDYKYRPRRKP 77

Dm-Sox21b       61 MHMKEHPDYKYRPRRKP 77

Tc-D            61 LHMKQHPDYKYRPRRKP 77

Pt-Sox14-like X 61 LHMKEHPDYKYKPRRKP 77

Pt-Sox21-like X 61 EHLRLYPDYKYRPRRKP 77
