# Supplementary material for: Embryonic expression patterns and phylogenetic analysis of panarthropod sox genes: insight into nervous system development, segmentation and gonadogenesis
Source: BMC Evol Biol. 2018 Jun 8;18:88. doi: 10.1186/s12862-018-1196-z (PMC5994082; doi:10.1186/s12862-018-1196-z)
Supplement: Supplementary file 4 — Whole-mount in-situ hybridization protocol. (DOCX 69 kb) [file 12862_2018_1196_MOESM4_ESM.docx]

**Whole mount in-situ hybridization (*Parasteatoda*, *Tribolium, Glomeris* and *Euperipatoides*)**

**_______________________________________________________________________day 1**

**Rehydration and post-fixation**

❑ Return embryos to RT

❑ Wash 5 min in 50% MeOH in PBS-T

❑ Wash 5 min in 30% MeOH in PBS-T

❑ ❑ ❑ Wash three times in PBS-T for 10 min each

❑ Fix for 20 min in 5% FA in PBS-T (1 ml PBS-T + 170 μl 37% FA)

❑ ❑ ❑ Wash three times in PBS-T for 5 min each

**Prehybridization**

❑ Wash embryos once in 1:1 PBS-T:HYB-B for 15 min (first add 500µl PBST, then 500µl HYB-B (mix gently by inverting)

❑ Replace and incubate embryos in 500μl HYB-B for 5 min @ 65°C

❑ Replace HYB-B with 500μl of prewarmed HYB-A

❑ Prehybridize @ 65°C for 2h in HYB-A

❑ Heat probes to 95°C for 2 min, then place on ice for 2 min

❑ Preheat probes to 65°C

**Hybridization**

❑ Remove HYB-A from the embryos without letting them touch air

❑ Add 50µl of prewarmed fresh HYB-A

❑ Add 2µL anti-sense RNA probe, mix gently, and incubate o/n @ 65°C (depending on quality and concentration of probe)

**________________________________________________________________day 2**

**Probe removal**

❑ ❑ Wash two times with 500μl prewarmed HYB-B, incubate 20 min @ 65°C

❑ Replace with 1:1 mixture of HYB-B:2XSSC incubate 30 min @ 65°C

❑ ❑ ❑ Wash three times with 2xSSC for 20 min each @ 65°C

❑ ❑ ❑ Wash three times with PBS-T for 10 min each @ RT

❑ Incubate in blocking buffer for at least 2 h @ RT

**Detection**

❑ Prepare 1:2000 dilution of AP conjugated DIG-antibody in 1 ml Blocking buffer

❑ Incubate embryos with DIG-antibody mixture for at least 1.5 h

❑ ❑ ❑ ❑ Wash four times in PBS-T for 10 min each

❑ Incubate in PBS-T o/n @ 4°C

**________________________________________________________________day 3**

**Staining**

❑ ❑ ❑ Wash three times with PBS-T for 5 min

❑ ❑ Wash two times with AP staining buffer for 10 min

❑ Incubate with BM purple staining solution and monitor color development under the microscope (stain in the dark)

❑ ❑ ❑ ❑ Stop staining reaction by washing at least 4 times with AP staining buffer (pH7.4) for 10 min each

❑ Counterstain with 2µl/ml DAPI for 30min (in the dark)

❑ ❑ ❑ Remove solution by 3 X washing for 10 min in STOP solution

❑ For long time storage, add 50μl 37% FA to 1 ml STOP solution (pH7.4) and store @ 4°C

**­­­10x PBS stock**

1.37 M NaCl

27 mM KCl

100 mM Na2HPO4

adjust pH to 7.4

autoclave

**PBS-T**

1 x PBS

0.02 % Tween-20

**HYB-B**

50% formamide

25% 20x SSC, pH 7.0

0.1% Tween-20

adjust pH to 6.5

**HYB-A**

1x HYB-B

0.05 mg/ml heparin

0.01 mg/ml yeast RNA

0.4 mg/ml sonicated salmon sperm DNA

Boil for 10 min and cool on ice for 3 min

store @ -20°C

**Blocking buffer**

1x PBS-T

1% BSA

2% Sheep serum

**AP staining buffer**

100 mM Tris pH 9.5

150 mM NaCl

10 mM MgCl2

0.1% Tween-20

**STOP solution**

Same as AP staining buffer, BUT ph 7.4
