# Supplementary figures and images for: Embryonic expression patterns and phylogenetic analysis of panarthropod sox genes: insight into nervous system development, segmentation and gonadogenesis
Source: BMC Evol Biol. 2018 Jun 8;18:88. doi: 10.1186/s12862-018-1196-z (PMC5994082; doi:10.1186/s12862-018-1196-z)

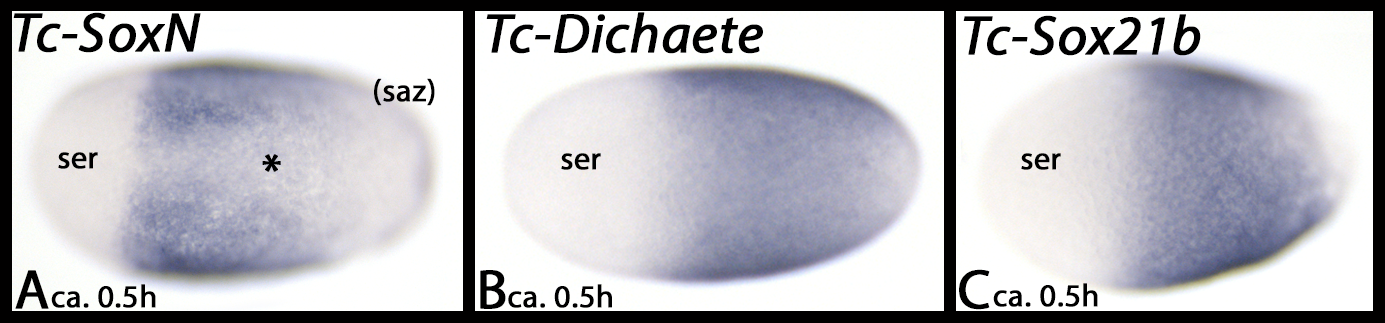

Supplement: Supplementary file 5 — Figure S2. Early expression of Tribolium SoxN, Dichaete and Sox21b. Anterior is to the left, ventral views. Developmental stages are indicated (after Strobl and Stelzer 2014). The asterisk (*) marks weaker expression in ventral tissue. Abbreviations: (saz) primordium of the segment addition zone; ser, serosa. (TIF 2612 kb) [file 12862_2018_1196_MOESM5_ESM.tif]

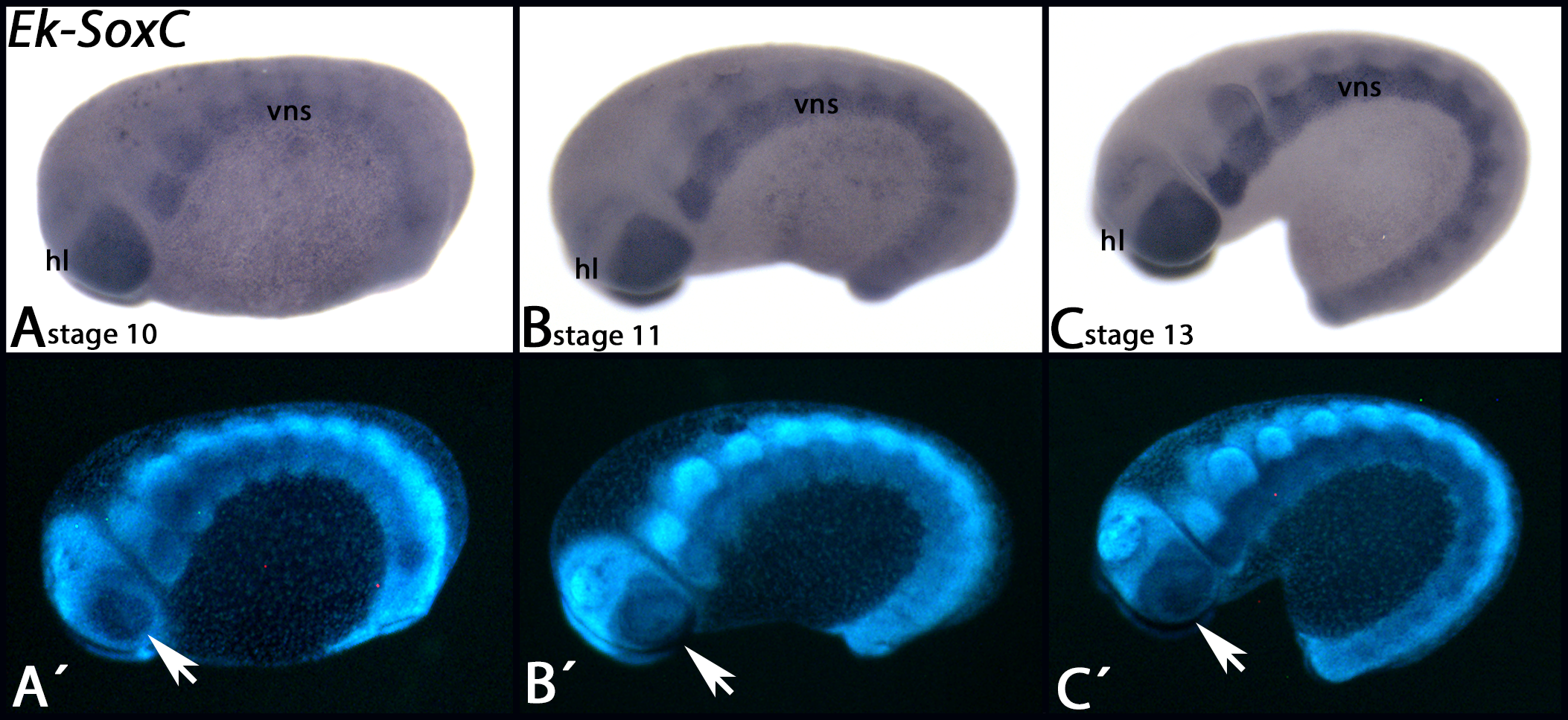

Supplement: Supplementary file 7 — Figure S3. Expression of Euperipatoides SoxC in the brain. Anterior is to the left, ventral views. Developmental stages are indicated (after Janssen and Budd [23]). Arrows point to dynamic expression in the brain (best seen in DAPI stained embryos). Abbreviations: hl, head lobe; vns, ventral nervous system. (TIF 10243 kb) [file 12862_2018_1196_MOESM7_ESM.tif]
