# Supplementary material for: Embryonic expression patterns and phylogenetic analysis of panarthropod sox genes: insight into nervous system development, segmentation and gonadogenesis
Source: BMC Evol Biol. 2018 Jun 8;18:88. doi: 10.1186/s12862-018-1196-z (PMC5994082; doi:10.1186/s12862-018-1196-z)
Supplement: Supplementary file 8 — Table S3. Overview expression data (DOCX 91 kb) [file 12862_2018_1196_MOESM8_ESM.docx]

|  | Hexapoda:  Diptera | Hexapoda:  Coleoptera | Hexapoda:  Hymenoptera | Myriapoda:  Diplopoda | Onychophora:  Peripatopsidae |
| --- | --- | --- | --- | --- | --- |
|  | ***Drosophila melanogaster*** | ***Tribolium***  ***castaneum*** | ***Apis***  ***mellifera*** | ***Glomeris***  ***marginata*** | ***Euperipatoides***  ***kanangrensis*** |
| *SoxNeuro* | brain, VNS, PNS, early head domain | brain, VNS | brain, VNS | brain, VNS | brain, VNS |
| *dichaete* | segmentation*, brain, VNS, PNS, hindgut | brain, VNS, SAZ, mandible | --- | brain, VNS, SAZ, mandible | brain, VNS, SAZ |
| *Sox21b* | --- | brain, VNS, SAZ, mandible | brain, VNS, mandible | --- | --- |
| *Sox21a* | foregut, hindgut, ventral midline | Malpighian tubules, ventral midline (weak), foregut/stomodaeum | Malpighian tubules, ventral midline (weak)*** | ???**** | --- |
| other Sox B genes | B2.3: no expression | *TcSoxB5*: no expression | *AmSox21*: no expression | --- | *EkSoxB3*: no expression |
|  |  |  |  |  |  |
| *SoxC* | ubiquitous | ubiquitous, brain, PNS(?) | ubiquitous, brain | ubiquitous, brain, VNS | ubiquitous, brain, VNS |
| *SoxD* | Brain, single cells in VNS | brain, segmental mesoderm, ventral midline | ubiquitous | ubiquitous, anal valves, limb mesoderm, dorsal mesoderm**, ventral midline | brain, VNS (salt-n-peppar), segmental stripes (mesoderm?), limb mesoderm |
| *SoxE* | gonad, alimentary canal, anal pad | gonad, Malpighian tubules; mesodermal derivatives (e.g. limb mesoderm) | E1/E2: no specific embryonic expression | E1/E2: anal valves, gonad, mesodermal derivatives (e.g. limb mesoderm) | blastopore, mesodermal derivatives (e.g. limb mesoderm) |
| *SoxF* | PNS | early SAZ, dorsal in gnathal segments, small domains in limbs including abdominal limb rudiments | ubiquitous | Labrum, ectodermal expression in appendages | anus (endoderm?), nephridial openings |

*PRG-like and SPG-like expression (cf. to expression in SAZ in short germ arthropods and the onychophoran)

**cf. Expression of *nautilus* (Janssen 2011)

*** (aka SoB2: Not described by Wilson and Dearden 2008, but visible in their Fig. 5B)).

**** A potential gene fragments was found that is too short for in-situ hybridization. RACE failed.
